# Measuring adult mortality from mobile phone surveys in Burkina Faso, Malawi and the Democratic Republic of the Congo

**DOI:** 10.1136/bmjgh-2025-019678

**Published:** 2025-11-21

**Authors:** Kassoum Dianou, Bruno Masquelier, Shammi Luhar, Bruno Lankoandé, Ashira Menashe-Oren, Abdramane Soura, Hervé Bassinga, Malebogo Tlhajoane, Boniface Dulani, Pierre Z Akilimali, Georges Reniers

**Affiliations:** 1 Institut Supérieur des Sciences de la Population (ISSP), Joseph Ki-Zerbo University, Ouagadougou, Burkina Faso; 2 Center for Demographic Research (DEMO), Louvain University, Louvain-la-Neuve, Belgium; 3 Department of Population Health, London School of Hygiene & Tropical Medicine (LSHTM), London, UK; 4 Department of Politics and Government, University of Malawi, Zomba, Malawi; 5 Kinshasa School of Public Health, University of Kinshasa, Kinshasa, Democratic Republic of the Congo

**Keywords:** Global Health, Health policies and all other topics, Maternal health, Demographic and Health Surveys, Data collection

## Abstract

In many low and middle-income countries, adult mortality estimates are derived from surveys and censuses conducted through face-to-face interviews. These interviews can be time-intensive and are often impractical during health crises or humanitarian emergencies. The expansion in cellphone ownership and network coverage has created new opportunities for collecting demographic data through mobile phone surveys, but our understanding of selection biases and reporting errors of such data remains incomplete. This study reports on adult mortality estimates obtained through mobile phone surveys conducted in Burkina Faso, Malawi and the Democratic Republic of the Congo in 2021 and 2022. To mitigate respondent fatigue and network interruptions, we used a shortened version of the set of questions generally used in surveys to ask about the survival of respondents’ siblings. Mortality estimates obtained from mobile phone interviews were lower than those from face-to-face demographic surveys. Mortality rates from the mobile phone surveys were also approximately half those expected from World Population Prospects (WPP) estimates. We attribute this underestimation primarily to reporting errors, including inaccuracies in the ages and timing of sibling deaths collected through the shortened instrument. Coverage biases due to mobile phone ownership likely played a secondary role in the reduced mortality estimates. After imputing ages and dates based on full sibling histories collected in previous face-to-face surveys, mortality rates were more consistent with WPP and Demographic and Health Survey estimates. However, estimates would be improved with more accurate age at death and time of death reports. Mobile phone surveys offer a promising alternative for monitoring adult mortality in settings where face-to-face data collection is not feasible, but they seem to be susceptible to more frequent reporting errors.

**WHAT IS ALREADY KNOWN ON THIS TOPIC**

- In many low- and middle-income countries, adult mortality estimates rely on survey-based methods such as sibling survival histories (SSH) collected through face-to-face interviews. These surveys are costly, time-consuming and difficult to conduct during crises. Additionally, the potential of mobile phone surveys (MPS) for measuring adult mortality remains underexplored.

**WHAT THIS STUDY ADDS**

- Based on surveys in Burkina Faso, Malawi and the Democratic Republic of the Congo, this study shows that SSH can be collected through mobile phone interviews.
- Summary data on the total number of adults and the number of those who died yield plausible mortality rates. However, mortality estimates from the MPS are lower than estimates based on face-to-face surveys.
- Estimates obtained from a shortened sibling history module collecting information on ages and dates appear to be biased downwards because of reporting errors, but imputation methods can help to partially correct these biases.

**HOW THIS STUDY MIGHT AFFECT RESEARCH, PRACTICE OR POLICY**

- Innovative methods for collecting adult mortality data in countries without comprehensive death registration systems are urgently needed, especially following the dismantling of the U.S. Agency for International Development (USAID) which had historically supported the Demographic and Health Surveys (DHS) programme. Our findings can help inform decisions on how to revive the collection of sibling survival data in this new demographic data landscape.
- The inclusion of questions on sibling survival in MPS can serve as a tool for mortality estimation that is complementary to the traditional, large-scale face-to-face surveys or can be an alternative when these surveys are impractical. Caution is warranted when using shortened versions of the questionnaires, as the data collected by phone on age at death and timing of deaths appear less precise than in standard DHS surveys.
- Alternative sets of questions, including the full SSH module with detailed questions on ages and dates, should be tested in pilot studies before being implemented in large-scale MPS.

## Introduction

 Comprehensive systems of death registration are the ideal source to obtain accurate and timely information on mortality and causes of death, but in many countries, these systems remain deficient.^1 2^ The completeness of death registration is particularly low in Sub-Saharan Africa, with the exception of a few countries, such as Zimbabwe and South Africa.^3^ In the absence of efficient systems of death registration, surveys and censuses remain the primary sources of mortality data. By pooling data sources together, it is now possible to monitor trends in child mortality with relatively good precision.^4^ Data are scarcer for adults. In particular, retrospective reports on the survival of siblings or parents collected in surveys are one of the most widely used data sources on adult mortality,^5^ alongside reports on recent household deaths collected in censuses. While these sources facilitate the reconstruction of mortality trends, data collection is usually organised through face-to-face interviews, which limits their potential for capturing rapid changes in mortality, as nationwide surveys or censuses are carried out irregularly. These large operations are also challenging during humanitarian and public health emergencies. For instance, during the Ebola and COVID-19 health crises, many survey programmes and census operations had to be temporarily suspended.^6^ More recently, the abrupt termination of the Demographic and Health Surveys (DHS) programme in 2025 following USAID’s dismantling has further eroded the already limited data on adult survival.

Alternative approaches to data collection are thus needed to monitor mortality more frequently, including in resource-constrained environments where in-person surveys are restricted. The expansion of mobile phone ownership and network coverage opens the door to mobile phone data collection in such contexts. Telephone interviews can be more cost-effective and offer greater flexibility in questionnaire design, quicker deployment, and consequently make it possible to collect data more frequently than traditional face-to-face surveys.^7^,^11^ Until recently, nationwide mobile phone surveys (MPS) were uncommon in low- and middle-income countries, but the COVID-19 pandemic greatly accelerated the adoption of MPS in these countries as well.^12^,^16^ So far, few MPS were designed to track changes in mortality. In Monrovia (Liberia), a study conducted by *Médecins Sans Frontières* showed that mobile phone data collection was a viable alternative for measuring excess mortality related to the Ebola crisis.^17^ Other MPS conducted in India have successfully documented excess mortality during the COVID-19 pandemic.^18 19^ These surveys were based on reports of household deaths, however, and experience is lacking around the feasibility of using other types of survey instruments in MPS, including sibling survival histories (SSH). In full SSH, respondents are asked about the survival status of each of their maternal siblings, their current age if alive and age at the time of death, as well as the time elapsed since death if the sibling is deceased.^20^ Although SSH are a well-established mortality measurement tool, this approach had never been tested within a MPS prior to this study.

Collecting sibling survival data over the phone presents several challenges. First, it may be desirable to keep questionnaires short, while collecting full SSH can take time in settings where fertility is high. Revisions to the standard SSH module might thus be required. Second, MPS could lead to more misunderstandings between interviewers and respondents, or lower commitment to the survey. A recent study in six Sub-Saharan African countries showed that age misreporting errors were more frequent in MPS than in recent household surveys and censuses.^21^ Third, respondents in telephone surveys may exhibit some reservations towards certain questions^22^ and be more hesitant to provide sensitive information about the deaths of their relatives over the phone. Encouragingly, a randomised trial in Malawi suggested that respondents in a telephone survey were as cooperative when asked about the mortality of relatives as when asked about their recent economic activity and that questions about the death of close relatives rarely generated negative reactions.^23^ Fourth, the representativeness of MPS respondents could be compromised, especially in places where mobile phone coverage is not universal.^724^,^26^ Respondents in MPS are more likely to be young, well-educated, urban and affluent, in part due to limited telephone coverage and lower purchasing power among poor populations.^7 26 27^ Stratified quota sampling and post-stratification weighting could help mitigate selection bias, but mortality rates may remain inaccurate if there is a causal link between mobile phone ownership and the survival of close relatives through, for example, improved care access and information.^25 28^

This study reports on SSH estimates of adult mortality from three MPS conducted in Burkina Faso, Malawi and the Democratic Republic of Congo (DRC). We used a shortened version of the standard SSH used in the DHS. We asked respondents to report on the total number of their maternal siblings, the number of such siblings who were still alive, and those who had died, with additional questions for those who had died since the beginning of 2019. Here, we first present an approach to imputing sibling histories collected with this shortened list of questions. We then evaluate the quality of the summary sibling data and compare adult mortality estimates obtained from the MPS to those from the World Population Prospects (WPP), as well as previous DHS and census data.

## Data and methods

### Data collection

The Rapid Mortality Mobile Phone Surveys Project (RaMMPS, https://www.lshtm.ac.uk/rammps/) is a multicountry survey programme to develop and apply methods for measuring mortality via MPS. In this study, we used data from the DRC, Malawi and Burkina Faso that included a SSH module. In Burkina Faso, the survey was coordinated by the *Institut Supérieur des Sciences de la Population* of Joseph Ki-Zerbo University; in DRC by the *Ecole de Santé Publique de l'Université de Kinshasa*; and in Malawi, it was a joint collaboration between the *Malawi Epidemiology and Interventions Research Unit* and the *Institute for Public Opinion Research*. All protocols received approval from both national health ethics committees and the LSHTM Research Ethics Committees (see declarations).

The characteristics of the three MPS are summarised in table 1. Interviews were conducted via mobile phone calls using the SurveyCTO application, which allowed both real-time data entry and automated call scheduling. Callback attempts were managed through SurveyCTO’s built-in scheduling system, following a predefined protocol. Each selected phone number was called at least one time, and those that did not pick up or were temporarily unreachable during initial attempts were called at least seven times, across different days and time slots. This approach aimed to maximise the likelihood of contact with all potential respondents. Following a few eligibility screening questions, participants provided verbal informed consent. Respondents were also informed that they would receive call credit on completing the interview to improve survey participation.^29^,^31^

**Table 1:** Characteristics of mobile phone surveys in the three countries covered

	Burkina Faso	DRC	Malawi
Data collection period	09/2021–10/2022	08/2021–08/2022	01/2022–07/2023
Sample size	21 339	11 924	14 663
Coverage	National	Kinshasa and North Kivu provinces	National
Recruitment strategy	Numbers based on a previous face-to-face survey (EHCVM) and random digit dialling	Random digit dialling	Random digit dialling
Target population for post-stratification	Population of heads of households (for the EHCVM arm) and population aged 15–64 (for random digit dialling) in the 2019 census	Population aged 18 and above in the MICS 2017–2018	Population aged 18 and above in the DHS 2015–2016
Study team	ISSP, Louvain University and LSHTM	UNIKIN and LSHTM	MEIRU, IPOR and LSHTM

DHS, Demographic and Health Survey; DRC, Democratic Republic of Congo; EHCVM, Enquête harmonisée sur les conditions de vie des ménages; ISSP, Institut Supérieur des Sciences de la Population of Joseph Ki-Zerbo University; LHSTM, London School of Hygiene & Tropical Medicine; MEIRU, Malawi Epidemiology and Interventions Research Unit; MICS, Multiple Indicator Cluster Survey; POR, Institute for Public Opinion Research; UNIKIN, Université de Kinshasa.

Across the three countries, we opted for different sampling designs and introduced variations in the questionnaires to assess respondent selectivity based on the recruitment approach (table 1). In Burkina Faso, data collection was carried out through quarterly cross-sectional surveys conducted over a 12-month period, to mitigate biases associated with seasonality of mortality. Two distinct sampling strategies were employed. The first subsample, referred to as the EHCVM arm, comprised approximately 6000 individuals selected based on telephone numbers collected during a prior face-to-face survey known as the Harmonized Household Living Conditions Survey (EHCVM for *Enquête Harmonisée sur les Conditions de Vie des Ménages*). For the MPS, we re-contacted each head of household who had been previously interviewed in the face-to-face EHCVM survey and provided a mobile phone number. Additionally, with the consent of the head of the household, a woman of reproductive age (15–49 years) residing in the household was randomly selected for inclusion. The second subsample, referred to as the RDD arm, included approximately 9000 individuals randomly selected by drawing telephone numbers through random digit dialling (RDD). We set quotas for a number of population strata to ensure the representativeness on age, gender and place of residence, using nationwide distributions based on the latest national census data.^32^ Numbers were randomly generated, accounting for the prefixes used by the major cell phone operators in the country (MoovAfrica, Telecel Faso and Orange). Numbers were screened by a company dialling each number to verify their functionality. More details on the Burkina Faso MPS are provided elsewhere.^33^

In DRC and Malawi, we only used RDD. In these two countries, we worked with a third party to randomly draw numbers based on the mobile phone structure used by the major operators in these countries. In the DRC, these were further restricted to numbers that were active in the provinces of Kinshasa and North Kivu in the recent past. As in Burkina Faso, we also resorted to quotas informed by national or provincial distribution of the population on a number of key attributes.

To reduce the length of the interviews, we developed a shortened version of the full SSH module used in DHS. The questions focused on the total number of siblings, the count of siblings currently alive and those who had passed away since the start of 2019. We collected supplementary details only for recent deaths, including the date, circumstances and location of the death (see online supplemental section A.4.1, table 2). The questionnaires for Malawi and DRC were less developed than in Burkina Faso on this module; no questions were asked about ages at death in DRC, and the data do not allow disaggregation between brothers and sisters in Malawi (online supplemental section A.4.1, table 3). In what follows, we therefore present results by sex for the first two countries, and results for both sexes combined in Malawi.

### Estimating adult mortality from MPS

#### Post-stratification weighting

As indicated earlier, mobile phone ownership is generally higher among individuals with higher socioeconomic status and education.^9 34^ To correct for this source of errors, we used post-stratification weighting.^7^ Weights were calculated using an iterative proportional fitting (a.k.a. raking) procedure to ensure that the marginal distribution of the weighting variables matched the distribution in a target population, such as the latest DHS or census (see table 1). The selection of target populations in our weighting process varied slightly by country due to the availability of variables and the use of different external sources (see online supplemental section A.4.1, table 4). In DRC, for example, we used the 2017–2018 Multiple Indicator Cluster Survey (MICS) as the external source to obtain the target distributions for age groups (18–39 and 40+), education level (non-primary, secondary and tertiary), a summary index of household asset ownership (based on whether the household had electricity, a durable roof and access to an improved water source), household size (1–5 and 5+ members) and urban/rural residence. Because we worked in two provinces only, we calculated separate sets of weights for Kinshasa and North Kivu and combined them to obtain final weights based on the proportion of the population in Kinshasa (63.5%) and North Kivu (36.5%) among the total population of these two provinces. To avoid high variability when using post-stratification weights, we limited the maximum weight to 2 in the three surveys.^35^

#### SSH

SSH are widely used for estimating adult mortality in low- and middle-income countries.^5^ Respondents are asked to list all children born to their biological mother, in birth order. For each sibling, information is collected on sex, survival status, current age (if alive) or age at death (if deceased), and, when applicable, the time elapsed since death. Additional questions are asked to identify pregnancy-related deaths. SSH offers many advantages, such as capturing a larger number of deaths and person-years than questions related to household deaths in the past 12 months. All the same, SSH has several well-documented limitations, including recall bias (especially for deaths that occurred in the distant past), age misreporting, omission of deceased siblings (particularly those who died in early adulthood or before the respondent was born) and displacement of death dates to avoid sensitive topics or due to poor memory.^5 20 36^ Such biases can lead to the underestimation of adult mortality. When SSH is implemented via mobile phone surveys, these limitations may be exacerbated. The abbreviated interview format, lack of visual cues and potential distractions during phone calls may reduce respondents’ ability to recall and report detailed sibling histories accurately. In addition, shorter question modules may leave less room for probing or clarifying inconsistent responses. These constraints can lead to further omissions and misclassifications, which may contribute to the underestimation of mortality levels.

#### Imputation to obtain full SSH

The full SSH module used in DHS, with questions on ages at the survey or at death and the timing of deaths, makes it possible to estimate adult mortality rates directly. Because both the occurrence of death and the exposure time (ie, the age and duration lived) are recorded or can be derived, mortality rates can be calculated by dividing the total number of deaths observed in a specific age group by the total person-years lived by all siblings in that age group, yielding an age-specific mortality rate.^37^ In our case, as we did not know the age at the time of the interview for surviving siblings and the age at death for deceased siblings who died before 2019, we could not estimate mortality directly and had to resort to imputation or modelling. Drawing from methods used by Hill *et al* to impute summary birth histories,^38^ we imputed ages and dates of death from an earlier DHS survey onto the summary sibling data collected in the MPS. We used the 2021 DHS in Burkina Faso, the 2015–2016 DHS in Malawi and the 2013–2014 DHS in the DRC.

Two approaches were used, referred to as *partial* and *complete* imputation in what follows. With *partial imputation*, we used all the information provided by respondents regarding the ages at death and the dates of death of recently deceased siblings, and we only imputed the age at the time of the survey for the surviving siblings, as this information was not collected. To achieve this, we randomly drew an age difference between each respondent and their surviving siblings from distributions found in the DHS, tabulated for each 5-year age group of respondents, and in Burkina Faso and the DRC, by the sex of the sibling. Using the age difference and the respondent’s age, we recalculated the age of all surviving siblings at the time of the survey.

For the *complete* imputation, we also imputed an age of death and a date of death for the deceased siblings, although this information had been collected for those who died recently. We cross-tabulated the siblings’ age at death and time since death from the DHS, categorised by the 5-year age group of respondents and in Burkina Faso and DRC by sex of the siblings. Based on these matrices, we proportionally sampled an age at death and a time since death for each deceased sibling. From the imputed values, we could then calculate the date of death and infer the date of birth for deceased siblings. With the *complete* imputation, we thus discard all the information reported in the MPS on ages and dates for recent deaths and keep only the number of siblings ever born and deceased, combining these summary counts with data from the previous DHS to impute all ages and dates.

**Figure 1:**
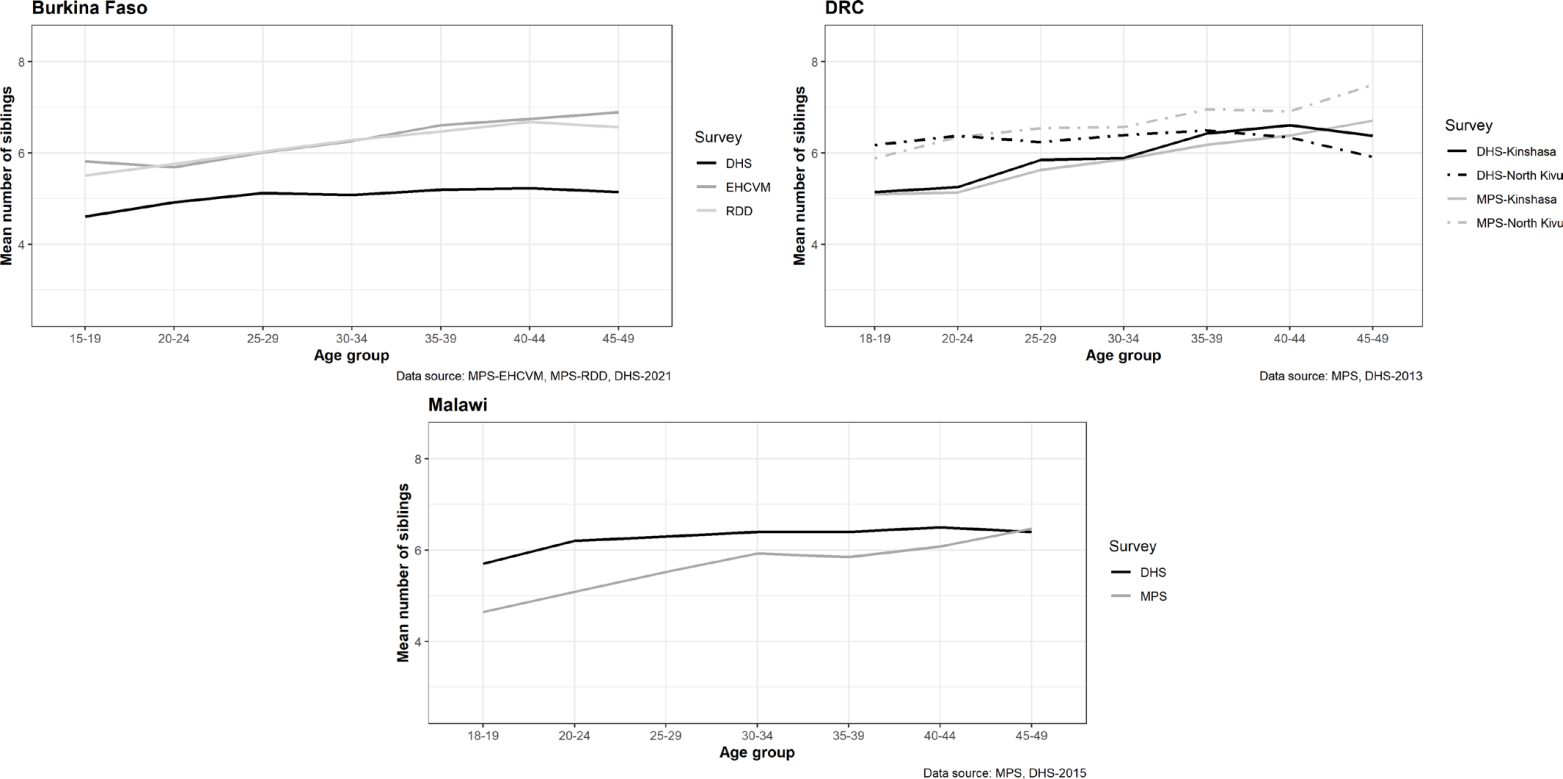
Mean number of maternal siblings by age group of respondent in the MPS and DHS conducted in Burkina Faso, Malawi and DRC. Note: In Malawi and the DRC, the MPS samples were restricted to ages 18 and above, and we also excluded respondents aged 15-17 in the DHS samples. DHS, Demographic and Health Surveys; DRC, Democratic Republic of Congo; EHCVM, Enquête harmonisée sur les conditions de vie des ménages; MPS, mobile phone surveys; RDD, random digit dialling.

**Figure 2:**
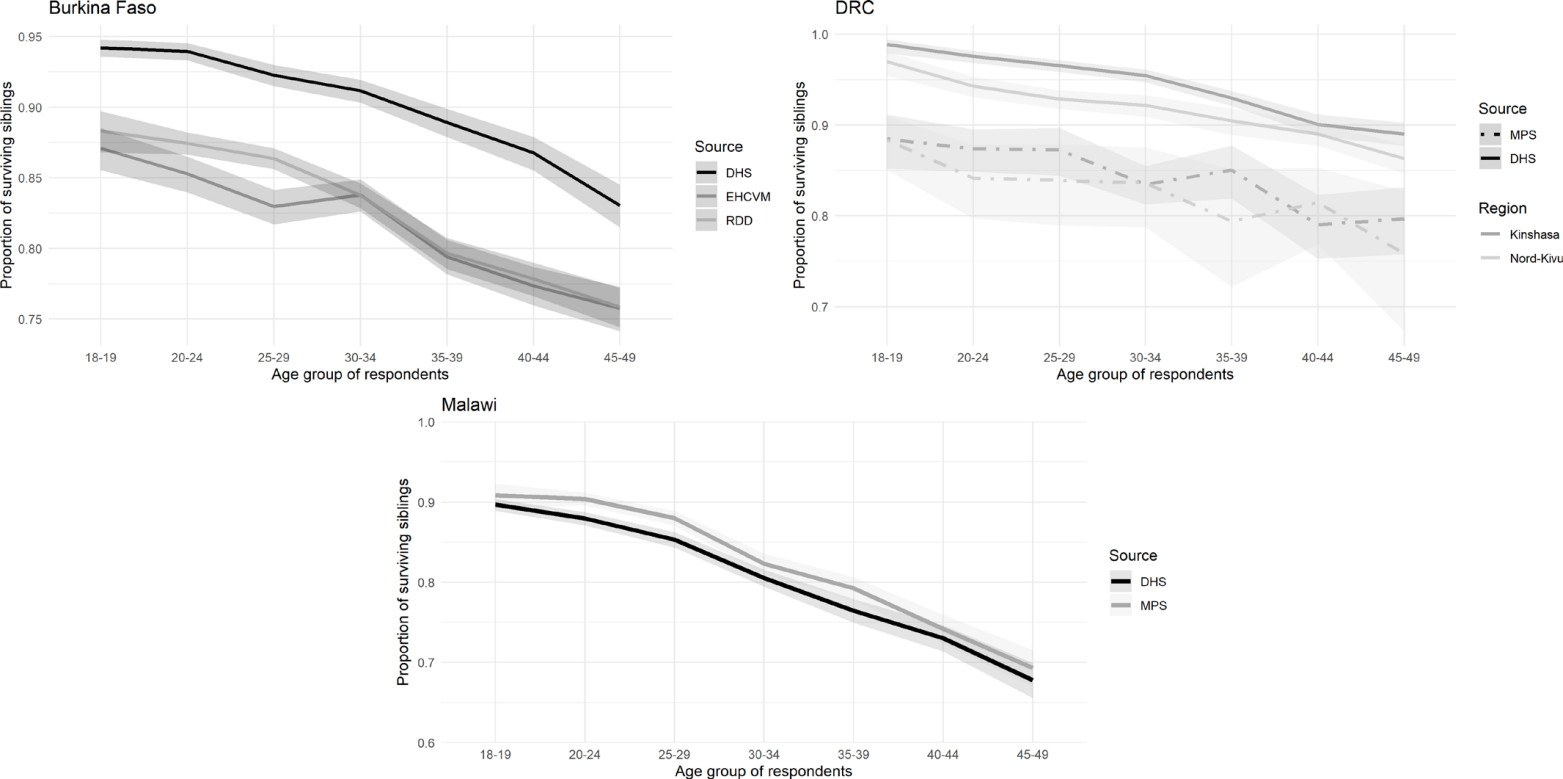
Proportions of surviving siblings at the time of the survey, by age group of respondent in MPS and DHS, in the three countries. DHS, Demographic and Health Surveys; DRC, Democratic Republic of Congo; EHCVM, Enquête harmonisée sur les conditions de vie des ménages; MPS, mobile phone surveys; RDD, random digit dialling.

**Figure 3:**
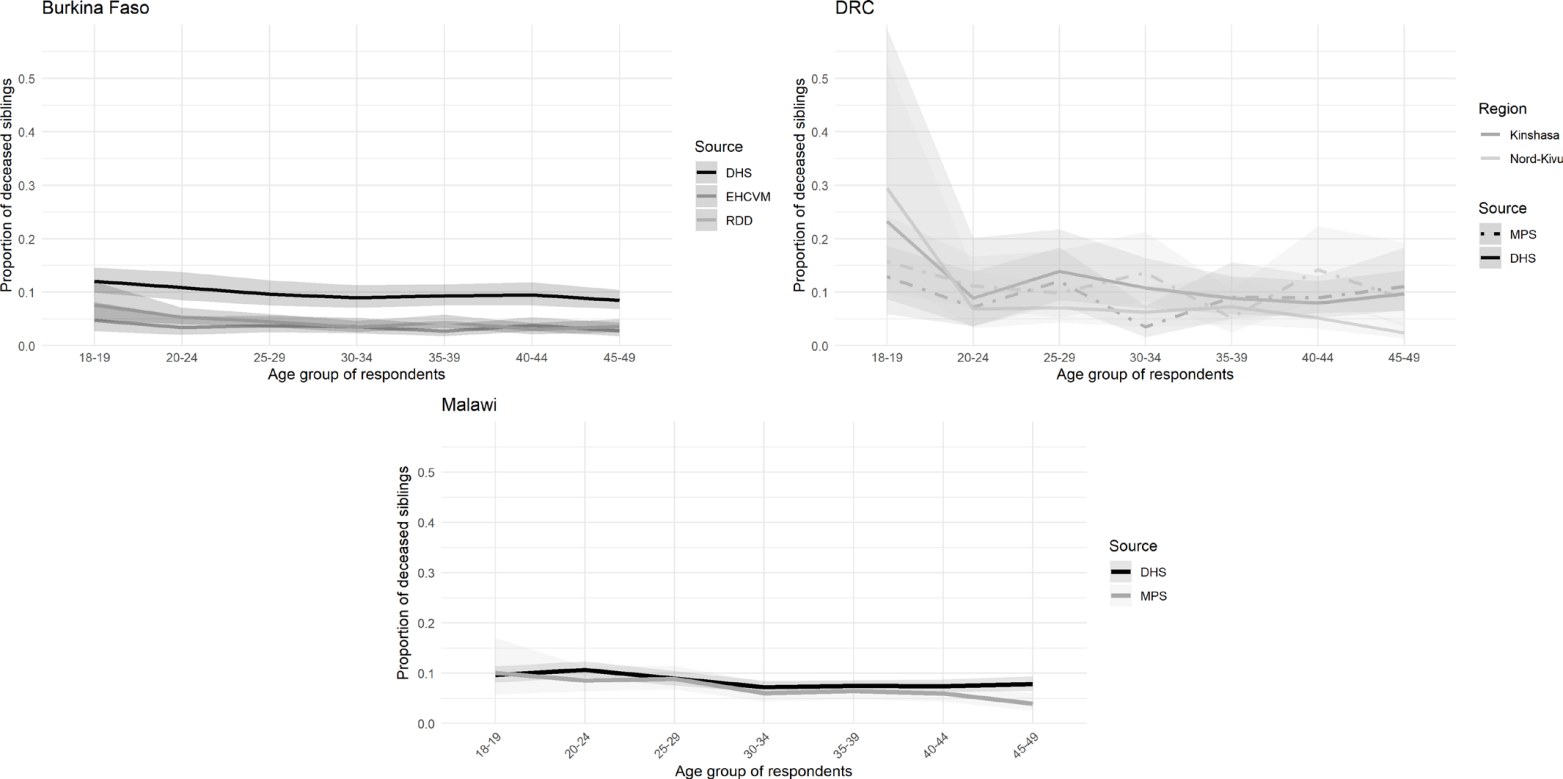
Proportions of recent deaths among all deceased siblings by age group and country (last 3 years in DHS and since January 2019 in MPS). DHS, Demographic and Health Surveys; DRC, Democratic Republic of Congo; EHCVM, Enquête harmonisée sur les conditions de vie des ménages; MPS, mobile phone surveys; RDD, random digit dialling.

To ensure that our two imputation approaches produced acceptable results, we tested them on existing DHS where at least two surveys had included a module on sibling mortality (82 DHS from 41 countries, listed in online supplemental section A.1 figures 1–3 and table 1). For each country, we retained the most recent DHS and applied both the *partial* and *complete* imputation approaches, using the preceding DHS to draw ages and times since death. We calculated mortality rates from the imputed sibling histories and compared the results with rates obtained directly from the reported ages and dates in the last survey. The results of this test are detailed in supplementary materials (online supplemental section A.1). The *partial* imputation provides results that are very close to the direct estimates: the mean absolute percentage deviation between the imputed and reference estimates is lower than 2.5% for each sex. The *complete* imputation is less precise, and the relative deviations are quite large: the mean absolute percentage deviation is 23% for each sex. The median ratio of imputed to reference estimates is 0.89 for men and 0.96 for women, indicating that this approach tends to produce underestimates, except for a few countries. Still, in Burkina Faso, Malawi and the DRC, both methods give acceptable results. In these three countries, we also have a relatively recent DHS to impute data onto the age-specific distributions of the total number of siblings and deceased siblings of the MPS.

#### Obtaining age-specific mortality and the probability _35_q_15_

After expanding the summary SSH collected in MPS to a full SSH through imputation, we estimated adult mortality using a direct method, by dividing the deaths by the corresponding exposure times, by age and by period since data collection. We converted the mortality rates into survival probabilities (assuming that deaths occur halfway through 5-year intervals), chained the survival probabilities together and converted these cumulative probabilities into _35_q_15_, the probability that an adolescent aged 15 years would die before reaching age 50 years. We used the ‘demogsurv’ package of the statistical software R (https://github.com/mrc-ide/demogsurv). CIs around the probability _35_q_15_ were obtained through the jackknife method. All estimates were weighted using individual sampling weights that account for the survey design and ensure population-level representativeness. The probability _35_q_15_ was retained as this is a common summary measure of adult mortality.

## Results

### Characteristics of the MPS samples

The characteristics of the MPS respondents differ systematically from those of the reference national populations. This is detailed in the supplementary materials (see online supplemental figure 4 and tables 5–7). The reference populations are based on the 2019–2020 census for Burkina Faso, the 2018 census for Malawi and the 2017–2018 MICS survey for the DRC.

In the three MPS, there is an under-representation of rural respondents. For instance, in Burkina Faso, rural respondents constitute approximately 43% and 63% of the EHCVM and RDD samples, respectively, while they account for 69% of household heads in the census (rural adults aged 15 to 64 also account for 69% of adults of the same age in the census). In Malawi, rural respondents represent 74% of the surveyed population in MPS, compared with 83% of the population enumerated in the 2018 census. The population with higher levels of education is also over-represented in the MPS. Finally, all MPS are characterised by the under-representation of respondents living in small households. The difference is striking in Malawi, where 39% of MPS respondents live in households with one to four members, against 92% in the 2019 census.

After applying post-stratification weights, our three samples have characteristics that are comparable to our reference populations, with some exceptions (see online supplemental tables 6–8). In all three surveys, members of larger households remain over-represented even after weighting. In the DRC, we also retain an over-representation of men and of the urban population in North Kivu.

### Assessment of the quality of the MPS data on summary sibling histories

To evaluate the quality of data on siblings, we first examined the mean number of reported siblings and compared each MPS with the last DHS survey that included a module on adult mortality (figure 1). The number of siblings is expected to be slightly lower in the MPS than in the DHS conducted a few years earlier, as a result of the fertility decline. In Burkina Faso, the two surveys were carried out only a year apart, but participants in the MPS tended to report a higher number of siblings, irrespective of the study arm. By contrast, in Malawi, the MPS shows the expected increase in mean number of siblings as the age of respondents rises, but with fewer siblings reported than in the 2015 DHS (up to one less sibling in the MPS in respondents aged 15–19). In DRC, there is virtually no difference between the two data sources in Kinshasa, but in North Kivu, respondents in the MPS, particularly the older age groups, reported slightly more siblings. However, these differences remain relatively small, averaging less than 0.5 siblings.

In figure 2, we present the proportions of surviving siblings categorised by the age group of respondents in each MPS, again comparing with the latest DHS with full sibling histories (see online supplemental figure 5 for sex-specific results). As expected, there is a consistent decline in the proportion of surviving siblings as the age of the respondent increases, regardless of the data source. However, there are significant differences between the data series. In Burkina Faso, proportions of surviving siblings from both MPS study arms are lower than in the 2021 DHS, while the reverse is true in DRC in both provinces. In Malawi, there is a good agreement between the MPS and the 2015 DHS.

Figure 3 presents the proportion of deaths that occurred in the 3 years preceding the survey, classified by age group of the respondent. We again compare these proportions to those calculated in the most recent DHS (see online supplemental figure 6 for sex-specific results). Regardless of the country, the proportions of recent deaths among siblings who have passed away are lower in the MPS than in the DHS, except for sisters in the Kinshasa province. While these disparities are relatively small in absolute terms, they have important implications for estimating recent mortality, as even modest biases in age or date reporting can lead to significant distortions in mortality estimates, particularly when using truncated data to reconstruct recent trends. The smaller percentage of recent deaths observed in MPS suggests that some recent deaths were omitted or shifted backwards in time and will not contribute to mortality estimation in the last 3 years.

### Adult mortality rates in MPS based on partial imputation

**Figure 4:**
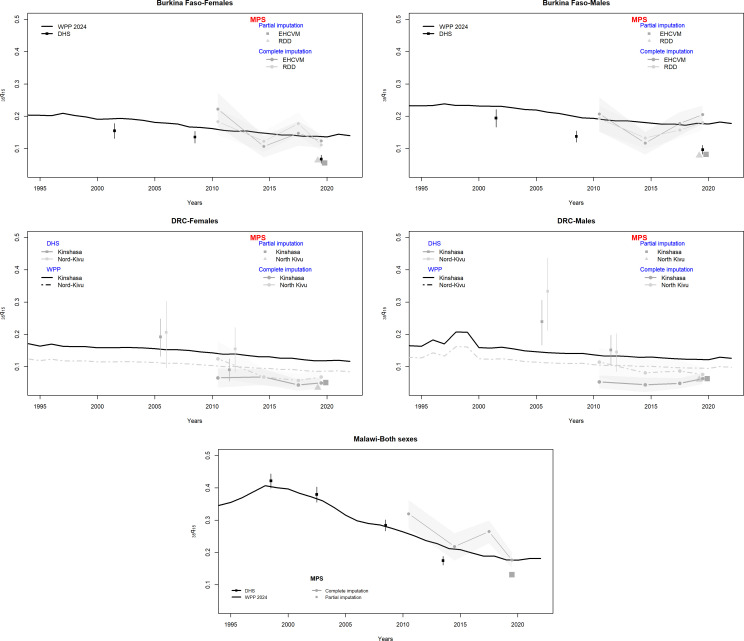
Trends in adult mortality [35q15] according to SSH in the MPS survey or DHS surveys and in the World Population Prospects (WPP). DHS, Demographic and Health Surveys; DRC, Democratic Republic of Congo; EHCVM, Enquête harmonisée sur les conditions de vie des ménages; MPS, mobile phone surveys; RDD, random digit dialling; SSH, sibling survival histories.

Figure 4 presents trends in the synthetic cohort probability that a 15-year-old adolescent dies before reaching age 50 (_35_q_15_), when using all the data provided by respondents on ages and the timing of death, and imputing only the age of surviving siblings. To put the MPS estimates in perspective, we compare them to those extracted from the most recent DHS. The DHS estimates are based on full sibling histories and are also calculated for the 0–3-year period preceding data collection. We also present estimates from the WPP, using the 2024 Revision. These draw on a multitude of sources, including sibling survival data, parental survival information, recent household deaths and model age patterns of mortality.^39^ The WPP estimates are available at the national level only, and to obtain reference adult mortality rates for DRC for the two provinces, we scaled the national adult mortality rates in the WPP according to the ratio of province-specific to national under-five mortality in the latest DHS (see online supplemental appendix A.2 for more details). It is worth noting that the WPP estimates are usually higher than estimates from SSH collected in surveys, and this could be due to underestimation of mortality in SSH or, in some countries, an upward bias in the WPP.^40^ Online supplemental table 8 provides the estimates by sex.

Overall, the MPS estimates of adult mortality are lower than those in the latest DHS. In Burkina Faso, the MPS estimate of _35_q_15_ (both sexes) is 14% lower. In Malawi, _35_q_15_ is 25% lower than the estimate extracted from the 2015 DHS. It is, however, possible that adult mortality declined rapidly in-between the two surveys, for example, thanks to the scale-up of antiretroviral therapy. In DRC, the differences with the previous DHS are much larger (−53% in Kinshasa and −68% in North Kivu), but in this country, the comparison is more complicated as estimates refer to subnational areas.

MPS estimates are also considerably lower than those reported in the WPP. In Burkina Faso, for example, the WPP estimate of the probability that a 15-year-old would die before their 50th birthday in 2019 was 178 and 138 per 1000 for men and women, respectively. In contrast, the MPS yielded estimates of 81 and 60 per 1000 for men and women. This translates into a downward bias in the _35_q_15_ probability of 54% for men and 57% for women. The deviations are similar in the Kinshasa province of DRC (−54% for male mortality and −56% for female mortality), and slightly smaller in North Kivu (−31% and −59%, respectively). In Malawi, there is a better agreement across sources, but the _35_q_15_ estimate for both sexes combined is still 25% lower than the corresponding probability in the WPP.

Annual estimates in the probability _35_q_15_ are also presented in the appendix (online supplemental figure 7), showing fluctuations in mortality, but no evidence of a spike in mortality associated with the COVID-19 pandemic.

In Burkina Faso, the sex of the respondent or the sex of siblings does not appear to be systematically associated with deviations from the most recent DHS or WPP. For example, in the RDD arm, male respondents provide lower estimates than female respondents, but the reverse is true in the EHCVM arm (online supplemental table 8). No systematic pattern emerges in the DRC either. In Malawi, mortality rates extracted from male respondents are 37% higher than those based on female respondents, but the difference is not significant. An earlier study comparing reports on sibling survival according to the sex of respondents in 10 DHS did not detect significant or systematic differences in mortality estimates obtained from men and women either.^41^

### Adult mortality rates in MPS based on complete imputation

Disparities in adult mortality levels observed between the MPS (where we used a shortened questionnaire) and the DHS (based on full sibling histories) could be caused by misreporting ages and dates of death. To address this, we resorted to the *complete* imputation of all ages and dates of death of siblings and compared the resulting estimates again to the DHS and WPP (figure 4 and online supplemental table 9).

The MPS estimates obtained after complete imputation are higher than those based on the partially imputed data. In Burkina Faso, the _35_q_15_ estimate from the MPS is now 85% higher than extracted from the 2021 DHS, while in Malawi, our more recent estimate is virtually the same as the probability estimated from the DHS 2015 (+1%). By contrast, in the DRC, they remain lower than the 2013 DHS, with relative differences exceeding 50%, regardless of the province.

MPS estimates based on complete imputation in Burkina Faso and Malawi closely align with those from the WPP. For instance, in Burkina Faso in 2019, the WPP estimated that, out of 1000 individuals aged 15, 158 would die before reaching age 50, while the corresponding MPS estimate is 154 per 1000 (-3% only). In Malawi, the relative difference is only 1% for both sexes combined. In contrast, in the DRC, regardless of the province, MPS estimates are still well below the reference values from the WPP (−20% and −53% for North Kivu and Kinshasa, respectively).

A breakdown by study arm in Burkina Faso shows that the EHCVM provided slightly higher estimates (165‱ (95% CI 148 to 183)), compared with 148‱ (95% CI 132 to 163) in the RDD arm (see online supplemental table 9). A breakdown by the sex of the respondent shows that in Burkina Faso and Malawi, estimates based on the complete imputation are higher when obtained from female respondents than from male respondents, regardless of the gender of the siblings. In the DRC, no systematic pattern emerges according to the sex of respondents.

## Discussion

In this study, we analysed the quality of data collected in MPS on sibling survival and assessed the plausibility of the resulting mortality estimates against the mortality levels expected from the DHS and WPP in Burkina Faso, Malawi and the DRC.

In comparison to the SSH module used in DHS, the MPS included a shortened version of the set of questions, and we developed two imputation approaches to reconstruct full sibling histories. To assess the validity of these imputation approaches, we first tested them on existing data from 82 DHS. Our results suggest that imputing only the ages of surviving siblings from an earlier survey yields unbiased estimates. In our test with existing DHS, all the imputed estimates fell within the 95% CIs around the direct estimates obtained from the reported ages. In contrast, imputing all ages and dates yielded less precise estimates, especially when surveys were spaced far apart, or in countries where mortality trends have been irregular (eg, due to the HIV epidemic). The estimates based on the complete imputation fell outside the CIs around the reference estimates in about two-thirds of the surveys and tended to be biased downwards. This imputation approach is also likely to produce imprecise results when there are differences in the composition of the samples of successive surveys. This test on the existing DHS surveys thus suggests that it is preferable to collect information on ages at death and the timing of deaths, rather than relying on imputations.

Nonetheless, all three MPS underestimated adult mortality levels when relying on dates and ages gathered over the phone. The extent of this underestimation is difficult to establish as there is considerable uncertainty surrounding expected mortality rates among adults in these countries, due to the lack of death registration data. Despite this uncertainty, it is likely that the MPS underestimated adult mortality, because the rates based on the reported dates and ages at death were lower than those extracted from full sibling histories collected in previous face-to-face surveys.

It is possible that this pattern is in part attributable to the mode of data collection, due to selection biases related to the characteristics of mobile phone users and to more frequent misreporting errors. As regards the selection biases, our analyses revealed several significant differences between the composition of the phone survey samples and those of previous face-to-face surveys. Respondents living in rural areas and those with lower educational levels were under-represented in our MPS samples, particularly in DRC, where the difference was significant. A more detailed comparison, presented elsewhere, between participants in the MPS in Burkina Faso and all household heads who owned a mobile phone in the original face-to-face survey conducted in 2018–2019 (EHCVM) also showed that phone survey participants were generally younger, more educated, more frequently resided in urban areas and lived in larger households and lived in larger households with more amenities.^33^ These findings align with recent work in other low- and middle-income countries.^7 42^ While we accounted for these biases through post-stratification weighting, it is possible that our weighting adjustments do not entirely remove all selection biases. Although our post-stratification weighting using iterative proportional fitting improved the alignment of key demographic variables with those of the reference population, some differences remained. A recent study based on mobile phone owners in DHS suggested that post-stratification weighting of sibling histories based on respondents’ background characteristics did not always produce a correction in the expected direction and needs to be used with caution.^28^ In our study, the mortality estimates may not sufficiently reflect the poorest segments of the adult population, those with lower levels of educational attainment and rural individuals.

The downward bias could also be a consequence of the use of a shorter series of questions, offering fewer opportunities to correct statements and giving rise to more confusion among respondents. This could have led to omissions of deaths or transfers of some recent deaths out of the reference period (2019–2022). These transfers would explain the lower proportion of recent deaths reported in the MPS, as compared with the DHS. Supplementary questions were asked for recent deaths, such as those related to the circumstances of death. This might have prompted interviewers to move certain deaths out of the reference period to reduce their workload.^43^ But respondents themselves might have faced difficulties in dating recent sibling deaths with precision when sibling histories take the form of the small set of questions used in the MPS. Furthermore, data on ages at death appear to be of poorer quality when collected by telephone, an observation already made with regard to the age of the respondents themselves in several MPS.^21^ Online supplemental figure 8 shows the distribution of ages at death of adult siblings according to the type of survey: we can see a much stronger attraction to certain ages in the MPS surveys than in the DHS. In Burkina Faso, for example, the Myers’ blended index for age heaping is 10 for men and 17 for women in the 2021 DHS survey, compared with 23 and 30, respectively, in the MPS. Overall, misreporting of the timing of deaths and ages therefore seems to play a larger role than selection biases in explaining why MPS estimates of adult mortality are biased downward. When the information collected on ages and dates is discarded, and we resort to complete imputation, the resulting mortality estimates align more closely with expected levels. However, collecting only summary data on siblings is not a viable strategy. As we have seen with our test with DHS, the imputation method can yield uncertain results, and this procedure is based on fairly strong assumptions. For example, it assumes that the distributions of age differences and the timing of deaths that prevailed in the past still apply in the more recent period.

More research is needed on the exact set of questions and their wording to collect shortened summary sibling data. For example, asking about the total number of siblings who have reached age 15, those who have died and how many of these adult deaths occurred recently makes it possible to apply indirect estimation.^44 45^ Future MPS should also pilot the collection of full sibling histories to better evaluate the potential of this module to be associated with other interview modalities than face-to-face surveys. Even in the absence of a mortality gold standard, randomly assigning respondents to different types of questionnaires would shed light on response patterns. Additionally, an effective communication strategy informing the population about the data collection period and the objectives of the MPS could play a role in reducing interview time and enhancing respondent cooperation, thereby enhancing the quality of the data collected.

## Conclusion

Based on three surveys conducted in Burkina Faso, Malawi and the DRC, this study provides insights into the potential and limitations of using MPS to estimate adult mortality. While MPS offer a cost-effective and rapid alternative to traditional face-to-face surveys, our results show that mortality estimates derived from MPS are lower than those from DHS and WPP. These discrepancies are likely partly driven by coverage biases (due to differential mobile phone ownership), but reporting errors appear to play a more important role.

While MPS can be relatively easily implemented from a logistical standpoint and can provide timely data in crisis-affected or hard-to-reach areas, our findings suggest that they cannot yet be relied on to produce accurate adult mortality estimates across all settings in Sub-Saharan Africa. Further methodological research is needed to explore strategies to minimise coverage bias while also refining data collection survey instruments to improve respondent engagement and the overall data quality. MPS are a promising complementary tool in mortality surveillance, but their current susceptibility to coverage errors and, more significantly, reporting errors requires methodological improvements before their wider adoption.

## Supplementary material

10.1136/bmjgh-2025-019678 online supplemental file 1

## Data Availability

Data are available upon reasonable request.
