# Supplementary material for: Measuring adult mortality from mobile phone surveys in Burkina Faso, Malawi and the Democratic Republic of the Congo
Source: BMJ Glob Health. 2025 Nov 21;10(11):e019678. doi: 10.1136/bmjgh-2025-019678 (PMC12645625; doi:10.1136/bmjgh-2025-019678)

# Appendix

## A.1. Evaluation of the two imputation approaches on existing DHS in Sub-Saharan Africa

This section presents results related to the two imputation approaches applied to 82 DHS surveys from 41 countries.

Figure S1 depicts the male probability _35_q_15_ estimated directly from the full SSH in the latest DHS, compared to the probability obtained when we impute only the ages of surviving siblings from an earlier survey in the same country (referred to in the main text as “partial imputation”). The differences between the two sets of estimates are negligible (Table S1). Absolute differences vary from -5‱ in Guinea to 14‱ in Togo (for females). Relative differences vary from -3% in several surveys to 10% in Morocco (for men), and the mean absolute percentage deviation between the imputed and reference estimates is lower than 2.5% for each sex. In all DHS, the imputed estimate falls within the 95% confidence intervals around the direct estimate obtained from the reported ages.

In contrast, there are relatively large deviations between estimates calculated directly and those based on the complete imputation, when we discard all the information reported on ages and dates, keep only the number of siblings ever born and deceased, and impute the dates of birth and dates of death from a previous DHS (Figure S2). The median ratio of imputed to reference estimates is 0.89 for men and 0.96 for women, indicating that this approach tends to produce under-estimates, except for a few countries. Absolute differences range from -85‱ in Haiti to +138‱ in Zimbabwe (for men). Even if we account for the fact that both the reference and imputed estimates are affected by sampling errors, the relative deviations are quite large: the mean absolute percentage deviation is 23% for each sex. For male mortality, the estimates based on imputation fall outside the confidence intervals around the reference estimates in 27 surveys, representing about two-thirds of our sample. This is the case in 30 surveys for female mortality. There are several potential explanations for these discrepancies. Firstly, some surveys may have occurred at intervals that are too far apart, to the point where the structure of sibships or the level and age pattern of mortality of the previous survey no longer accurately reflects the experience captured in the more recent survey. This could be the case in South Africa, for example, where there is a gap of 20 years between successive surveys. In other countries, the disruptions introduced by the HIV/AIDS epidemic could play a role in these discrepancies; the full imputation works particularly badly in Namibia or Zimbabwe, where adult mortality has declined thanks to the scale-up of ART. It is also possible that differences between surveys in terms of data quality or sample composition lead to such deviations. Comparisons of the mean number of siblings reported per cohort of respondents are not always consistent, and in some cases, the numbers reported in the most recent survey are implausibly low when compared to the preceding survey (Figure S3).

Despite these limitations, the imputation method produces acceptable results in several countries. In the three countries in which we carried out the MPS, the mean absolute percentage deviation averages 17.7% for men and 12.7% for women in the DHS. In these three cases, we also have a relatively recent DHS to impute data from a full sibling history onto the age-specific distributions of the total number of siblings ever born and the number of deceased siblings reported in the MPS to generate mortality estimates.

Supplementary Figure 1*: Risk of dying in adulthood (_35_q_15_) in females, as obtained from full SSH in the latest DHS with sibling data (0-5 years prior to the survey), compared to levels based when imputing only the ages of surviving siblings from a preceding DHS (partial imputation)*

***
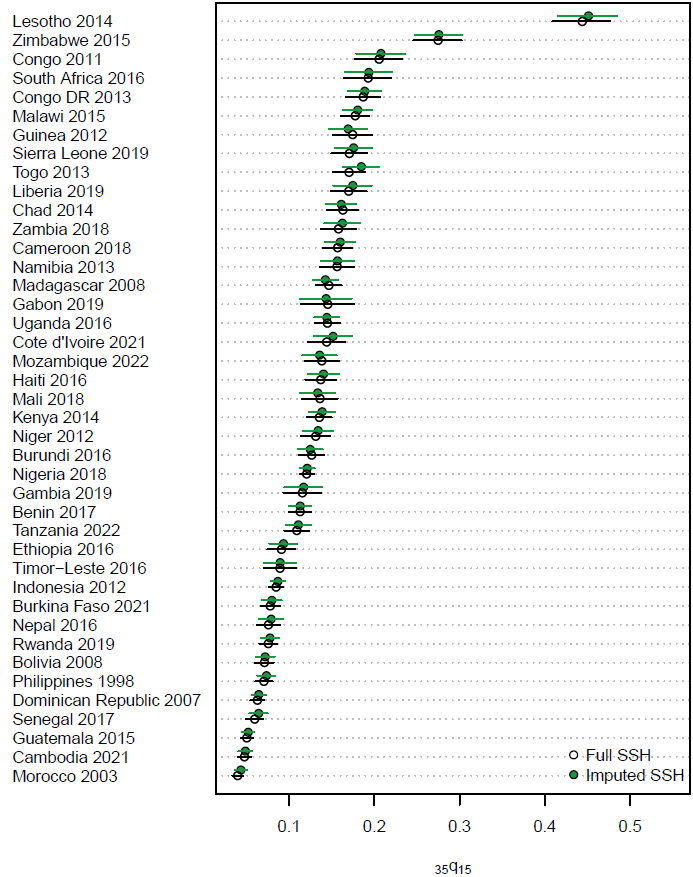
***

Supplementary Figure 2*: Risk of dying in adulthood (_35_q_15_) in females, as obtained from full SSH in the latest DHS with sibling data (0-5 years prior to the survey), compared to levels based when imputing all ages and dates of death from a preceding DHS* *(complete imputation)*

***
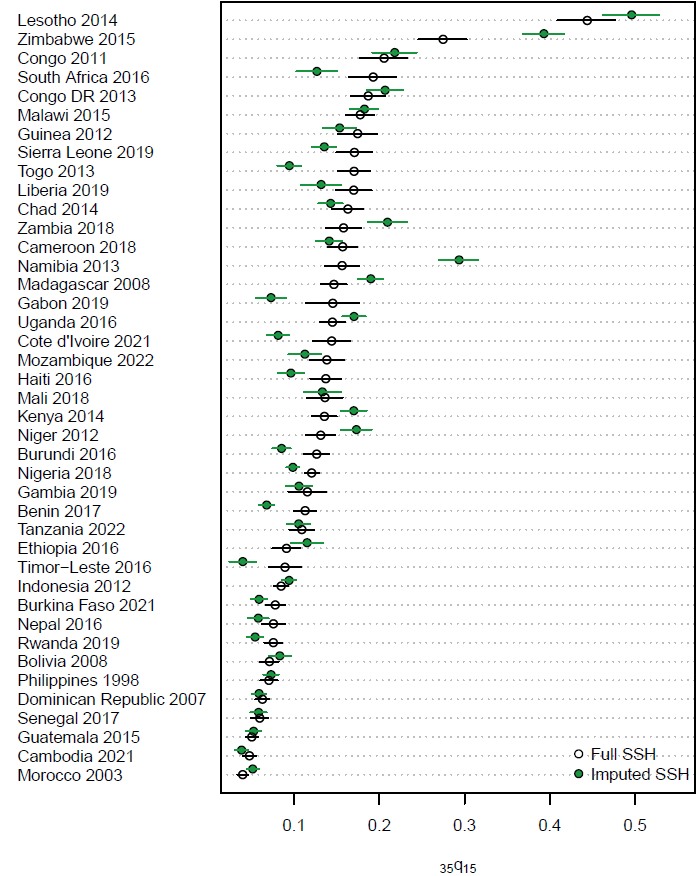
***

Supplementary Table 1*: Estimates of the probability _35_q_15_ estimated from the most recent DHS with sibling histories, without imputation and with two imputation approaches based on a previous DHS*

|  |  | Full SSH  Using all reported ages and dates | | | | Partial imputation  Imputation of ages of living siblings only | | | | Complete imputation  Imputation of all ages and dates of death | | | |
| --- | --- | --- | --- | --- | --- | --- | --- | --- | --- | --- | --- | --- | --- |
|  |  | Males | | Females | | Males | | Females | | Males | | Females | |
| Most recent DHS | Previous survey | _35_q_15_ | 95%CI | _35_q_15_ | 95%CI | _35_q_15_ | 95%CI | _35_q_15_ | 95%CI | _35_q_15_ | 95%CI | _35_q_15_ | 95%CI |
| Burkina Faso 2021 | 2010 | 0.104 | 0.092-0.116 | 0.078 | 0.067-0.089 | 0.107 | 0.094-0.12 | 0.080 | 0.068-0.091 | 0.067 | 0.057-0.076 | 0.059 | 0.049-0.069 |
| Benin 2017 | 2006 | 0.157 | 0.140-0.173 | 0.113 | 0.100-0.126 | 0.156 | 0.14-0.171 | 0.113 | 0.100-0.126 | 0.083 | 0.071-0.094 | 0.068 | 0.059-0.077 |
| Bolivia 2008 | 2003 | 0.087 | 0.075-0.099 | 0.071 | 0.060-0.082 | 0.089 | 0.077-0.101 | 0.072 | 0.061-0.083 | 0.098 | 0.086-0.110 | 0.083 | 0.070-0.096 |
| Burundi 2016 | 2010 | 0.182 | 0.165-0.199 | 0.127 | 0.111-0.142 | 0.181 | 0.164-0.197 | 0.125 | 0.110-0.139 | 0.134 | 0.120-0.149 | 0.085 | 0.075-0.096 |
| Congo DR 2013 | 2007 | 0.195 | 0.175-0.215 | 0.187 | 0.167-0.207 | 0.192 | 0.172-0.211 | 0.189 | 0.169-0.208 | 0.213 | 0.194-0.232 | 0.207 | 0.185-0.228 |
| Congo 2011 | 2005 | 0.177 | 0.149-0.204 | 0.206 | 0.177-0.233 | 0.19 | 0.159-0.219 | 0.208 | 0.179-0.236 | 0.184 | 0.159-0.208 | 0.218 | 0.192-0.244 |
| Cote d'Ivoire 2021 | 2012 | 0.15 | 0.128-0.171 | 0.144 | 0.123-0.165 | 0.161 | 0.137-0.184 | 0.152 | 0.129-0.174 | 0.073 | 0.059-0.088 | 0.081 | 0.068-0.094 |
| Cameroon 2018 | 2011 | 0.186 | 0.165-0.206 | 0.157 | 0.140-0.174 | 0.187 | 0.167-0.207 | 0.16 | 0.143-0.177 | 0.143 | 0.126-0.161 | 0.141 | 0.126-0.156 |
| Chad 2014 | 2004 | 0.190 | 0.169-0.210 | 0.163 | 0.145-0.181 | 0.189 | 0.169-0.209 | 0.161 | 0.143-0.179 | 0.156 | 0.139-0.172 | 0.143 | 0.129-0.157 |
| Dominican Republic 2007 | 2002 | 0.118 | 0.106-0.130 | 0.063 | 0.055-0.071 | 0.121 | 0.108-0.133 | 0.065 | 0.056-0.073 | 0.118 | 0.107-0.130 | 0.059 | 0.050-0.068 |
| Ethiopia 2016 | 2011 | 0.123 | 0.105-0.140 | 0.091 | 0.075-0.107 | 0.126 | 0.108-0.144 | 0.094 | 0.077-0.110 | 0.131 | 0.113-0.149 | 0.115 | 0.096-0.134 |
| Gabon 2019 | 2012 | 0.125 | 0.100-0.148 | 0.146 | 0.114-0.176 | 0.129 | 0.103-0.154 | 0.144 | 0.113-0.173 | 0.073 | 0.052-0.094 | 0.073 | 0.055-0.091 |
| Gambia 2019 | 2013 | 0.128 | 0.108-0.147 | 0.116 | 0.094-0.137 | 0.131 | 0.111-0.150 | 0.117 | 0.095-0.139 | 0.099 | 0.084-0.113 | 0.106 | 0.091-0.121 |
| Guinea 2012 | 2005 | 0.178 | 0.153-0.202 | 0.175 | 0.151-0.198 | 0.178 | 0.153-0.202 | 0.169 | 0.147-0.191 | 0.166 | 0.143-0.189 | 0.154 | 0.134-0.172 |
| Guatemala 2015 | 1995 | 0.111 | 0.101-0.121 | 0.051 | 0.044-0.057 | 0.115 | 0.105-0.125 | 0.052 | 0.045-0.059 | 0.091 | 0.081-0.102 | 0.053 | 0.044-0.061 |
| Haiti 2016 | 2006 | 0.171 | 0.150-0.192 | 0.137 | 0.119-0.155 | 0.171 | 0.151-0.191 | 0.140 | 0.122-0.158 | 0.086 | 0.071-0.102 | 0.096 | 0.081-0.111 |
| Indonesia 2012 | 2007 | 0.102 | 0.092-0.112 | 0.085 | 0.077-0.093 | 0.103 | 0.093-0.112 | 0.087 | 0.078-0.095 | 0.126 | 0.115-0.136 | 0.094 | 0.086-0.102 |
| Kenya 2014 | 2008 | 0.180 | 0.161-0.198 | 0.136 | 0.121-0.150 | 0.182 | 0.163-0.201 | 0.139 | 0.124-0.154 | 0.225 | 0.205-0.244 | 0.17 | 0.155-0.185 |
| Cambodia 2021 | 2014 | 0.105 | 0.092-0.117 | 0.048 | 0.040-0.056 | 0.107 | 0.094-0.119 | 0.049 | 0.041-0.057 | 0.06 | 0.052-0.068 | 0.039 | 0.031-0.046 |
| Liberia 2019 | 2013 | 0.198 | 0.168-0.228 | 0.170 | 0.149-0.191 | 0.206 | 0.174-0.237 | 0.175 | 0.152-0.197 | 0.133 | 0.110-0.155 | 0.132 | 0.108-0.155 |
| Lesotho 2014 | 2009 | 0.474 | 0.429-0.514 | 0.444 | 0.409-0.477 | 0.471 | 0.430-0.509 | 0.451 | 0.416-0.485 | 0.54 | 0.505-0.573 | 0.496 | 0.462-0.528 |
| Morocco 2003 | 1992 | 0.055 | 0.047-0.063 | 0.04 | 0.033-0.046 | 0.061 | 0.051-0.070 | 0.044 | 0.036-0.051 | 0.061 | 0.052-0.069 | 0.052 | 0.044-0.059 |
| Madagascar 2008 | 2004 | 0.158 | 0.144-0.172 | 0.147 | 0.132-0.162 | 0.154 | 0.139-0.168 | 0.143 | 0.128-0.158 | 0.234 | 0.216-0.251 | 0.19 | 0.175-0.205 |
| Mali 2018 | 2012 | 0.142 | 0.121-0.162 | 0.136 | 0.115-0.157 | 0.139 | 0.118-0.159 | 0.134 | 0.113-0.154 | 0.127 | 0.108-0.146 | 0.133 | 0.111-0.155 |
| Malawi 2015 | 2010 | 0.220 | 0.202-0.238 | 0.178 | 0.161-0.194 | 0.219 | 0.200-0.237 | 0.181 | 0.164-0.197 | 0.204 | 0.186-0.221 | 0.183 | 0.166-0.199 |
| Mozambique 2022 | 2011 | 0.174 | 0.146-0.201 | 0.139 | 0.118-0.158 | 0.173 | 0.144-0.200 | 0.136 | 0.116-0.156 | 0.132 | 0.113-0.150 | 0.113 | 0.093-0.132 |
| Nigeria 2018 | 2013 | 0.125 | 0.116-0.135 | 0.121 | 0.112-0.129 | 0.125 | 0.115-0.135 | 0.121 | 0.113-0.13 | 0.094 | 0.086-0.103 | 0.099 | 0.091-0.106 |
| Niger 2012 | 2006 | 0.139 | 0.119-0.158 | 0.131 | 0.114-0.148 | 0.138 | 0.119-0.157 | 0.134 | 0.116-0.152 | 0.142 | 0.122-0.160 | 0.173 | 0.155-0.191 |
| Namibia 2013 | 2006 | 0.242 | 0.217-0.267 | 0.156 | 0.136-0.176 | 0.251 | 0.225-0.275 | 0.157 | 0.137-0.176 | 0.347 | 0.317-0.377 | 0.294 | 0.270-0.316 |
| Nepal 2016 | 2006 | 0.093 | 0.077-0.109 | 0.076 | 0.062-0.089 | 0.099 | 0.081-0.116 | 0.079 | 0.065-0.093 | 0.073 | 0.058-0.088 | 0.058 | 0.046-0.070 |
| Philippines 1998 | 1993 | 0.133 | 0.119-0.146 | 0.071 | 0.060-0.081 | 0.136 | 0.122-0.150 | 0.074 | 0.063-0.084 | 0.143 | 0.131-0.156 | 0.073 | 0.064-0.082 |
| Rwanda 2019 | 2015 | 0.125 | 0.109-0.141 | 0.076 | 0.065-0.086 | 0.128 | 0.111-0.144 | 0.078 | 0.067-0.088 | 0.07 | 0.059-0.080 | 0.054 | 0.045-0.064 |
| Sierra Leone 2019 | 2013 | 0.207 | 0.186-0.227 | 0.171 | 0.149-0.192 | 0.208 | 0.188-0.228 | 0.176 | 0.154-0.197 | 0.134 | 0.118-0.149 | 0.136 | 0.121-0.150 |
| Senegal 2017 | 2010 | 0.073 | 0.061-0.085 | 0.060 | 0.050-0.070 | 0.078 | 0.065-0.090 | 0.064 | 0.054-0.075 | 0.072 | 0.06-0.084 | 0.058 | 0.049-0.068 |
| Togo 2013 | 1998 | 0.180 | 0.160-0.199 | 0.170 | 0.151-0.189 | 0.185 | 0.164-0.205 | 0.185 | 0.163-0.206 | 0.135 | 0.117-0.152 | 0.095 | 0.08-0.109 |
| Timor-Leste 2016 | 2009 | 0.109 | 0.083-0.133 | 0.089 | 0.070-0.108 | 0.106 | 0.082-0.129 | 0.09 | 0.070-0.109 | 0.039 | 0.025-0.053 | 0.040 | 0.024-0.056 |
| Tanzania 2022 | 2015 | 0.135 | 0.116-0.152 | 0.109 | 0.094-0.124 | 0.140 | 0.121-0.159 | 0.111 | 0.096-0.126 | 0.116 | 0.099-0.132 | 0.106 | 0.092-0.119 |
| Uganda 2016 | 2011 | 0.220 | 0.203-0.237 | 0.145 | 0.130-0.160 | 0.220 | 0.202-0.236 | 0.144 | 0.130-0.159 | 0.213 | 0.198-0.228 | 0.170 | 0.157-0.184 |
| South Africa 2016 | 1998 | 0.224 | 0.193-0.255 | 0.193 | 0.165-0.220 | 0.225 | 0.194-0.255 | 0.194 | 0.165-0.221 | 0.187 | 0.157-0.217 | 0.127 | 0.103-0.15 |
| Zambia 2018 | 2013 | 0.240 | 0.200-0.277 | 0.158 | 0.137-0.178 | 0.249 | 0.208-0.289 | 0.163 | 0.141-0.183 | 0.234 | 0.212-0.256 | 0.210 | 0.186-0.232 |
| Zimbabwe 2015 | 2010 | 0.302 | 0.274-0.329 | 0.275 | 0.246-0.303 | 0.304 | 0.277-0.331 | 0.276 | 0.248-0.303 | 0.440 | 0.412-0.467 | 0.393 | 0.368-0.417 |
|  |  |  |  |  |  |  |  |  |  |  |  |  |  |

Supplementary Figure 3*: Mean number of siblings ever born, tabulated by cohort of respondents, last two DHS surveys with SSH*


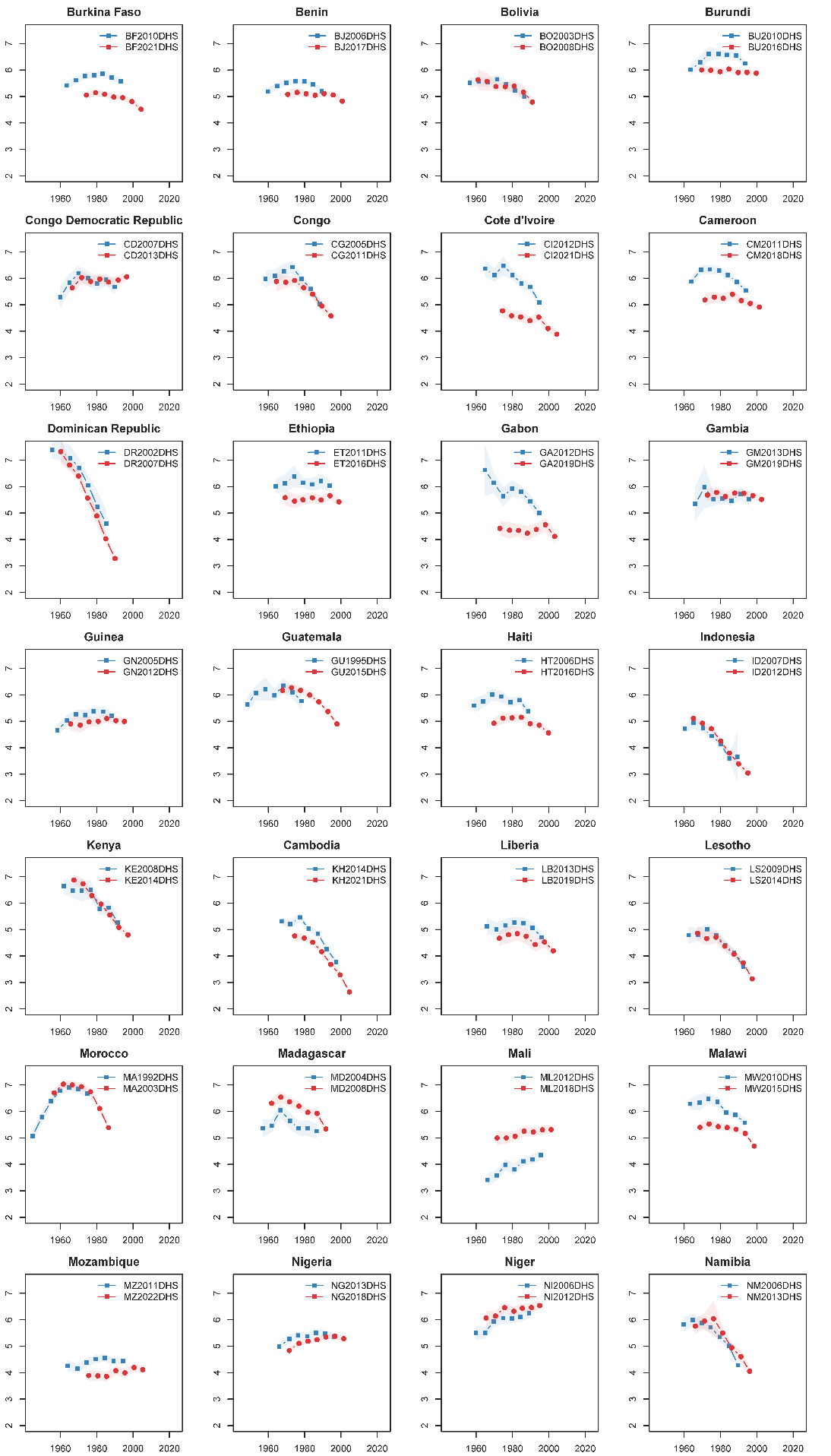


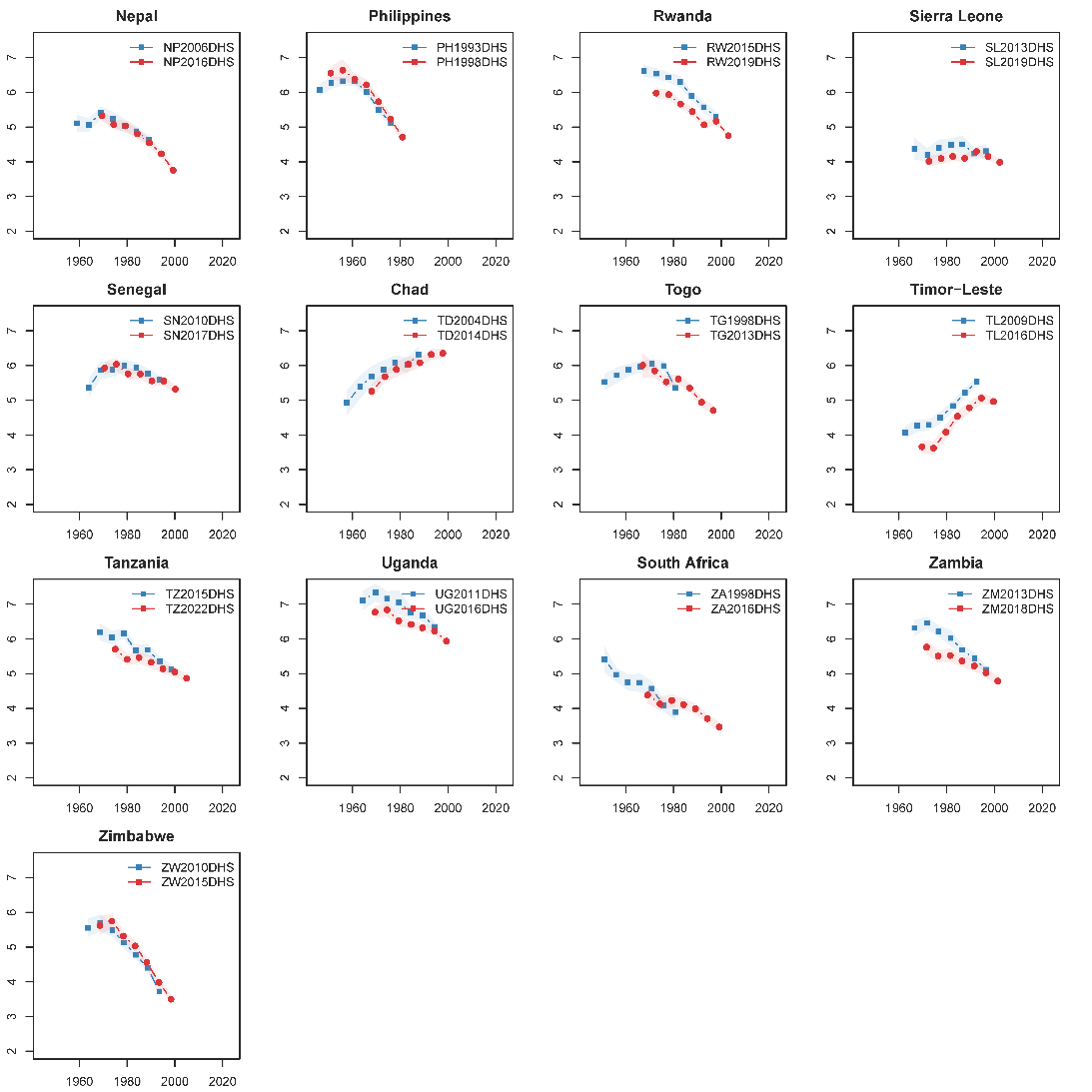


## A.2. Estimating sub-national mortality rates for the provinces of Kinshasa and North-Kivu

Because the adult mortality rates from the WPP refer to the national level, while the sibling-based estimates in DRC refer to two provinces only, we re-scaled the WPP estimates to obtain a reference for Kinshasa and North Kivu. We proceeded as follows:

(i) we used the Brass logit system (which has two parameters α – capturing the level of mortality – and β – capturing the age pattern) and assumed that β equals to 1;

(ii) we calculated under-five mortality for Kinshasa and North Kivu from the latest DHS conducted in DRC, and computed *Y(5)*, as the logit of survivorship, as follows:

Y(5) = -0.5 ln(l_5_/(1-l_5_).

(iii) we calculated the logits of the l_x_ values in the reference (national) life table from the WPP.

(iv) We estimated the adult mortality levels in Kinshasa and North Kivu, using the formula below;


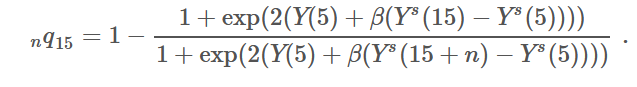


where β is set at 1, *Y(5)* refers to the logits of the DHS-based survivorship in childhood and *Y^s^(5), Y^s^(15)* and *Y^s^(15 + n)* refer to the logits of the survivorship for various age groups in the national WPP life table serving as our reference. This amounts to assuming that the age pattern of mortality in the WPP life table also applies to the two provinces, but the level of mortality is defined by the province-specific estimates of under-five mortality in the DHS.

## A.3 Post-stratification weighting procedure

Mobile phone ownership is strongly associated with socioeconomic status, education, and urban residence. Additionally, individuals living in larger households may be overrepresented due to a higher probability of answering calls. Post-stratification addresses these biases by aligning the sample with the true population structure. To improve the representativeness of the MPS data and reduce biases related to differential phone ownership and household composition, we applied post-stratification weighting using an **iterative proportional fitting procedure**, also known as **raking**. This method adjusts the marginal distributions of selected variables in the survey sample to match those of a reliable reference population (e.g., DHS, census, or MICS), while preserving the joint distributions observed in the MPS data.

#### **1. Selection of target population distributions**

Target population distributions were drawn from the most recent nationally representative surveys available for each country:

- **Burkina Faso**: 2019 General Census of Population and Housing (RGPH).
- **Democratic Republic of the Congo (DRC)**: 2017–18 MICS.
- **Malawi**: 2018 Population and Housing Census.

In DRC, region-specific population weights were computed and applied as the national source was not appropriate because the MPS was collected only in Kinshasa and North Kivu provinces.

#### **2. Variables used for raking**

The raking procedure was implemented to adjust for the following characteristics (depending on availability in both the MPS and the reference data source):

- **Age group** (e.g., 18–39, 40+)
- **Sex**
- **Education level** (none/primary incomplete, secondary, tertiary)
- **Household size** (1–5, 6+ members)
- **Urban/rural residence**
- **Asset index** (based on household access to electricity, improved water source, and durable roofing)

The selection of variables for each country was adapted based on the availability (see Supplementary Table 5 below).

#### **3. Raking implementation**

The raking algorithm was implemented in Stata using the ipfraking command, with the following steps:

1. Initial weights were set to 1 for all individuals.
2. Marginal distributions for each variable were iteratively adjusted to match the target population.
3. The process was repeated until convergence (defined as less than 0.01% difference between weighted sample margins and targets).

To **prevent extreme weights** (which can inflate variance and reduce precision), we **capped the maximum weight at 2**, a common threshold in survey analysis (DeBell, 2009). We verified that the weighted estimates remained robust and stable across sensitivity checks.

#### **4. Province-level weights (DRC example)**

In the DRC, where data were collected in two provinces (Kinshasa and North Kivu), we applied province-specific raking procedures. Final national-level weights were calculated by combining province-specific weights proportionally based on their estimated population share: 63.5% for Kinshasa and 36.5% for North Kivu.

## A.4 Additional tables and figures

### A.4.1 Additional tables

Supplementary Table 2*. Questions used to collect summary sibling histories in MPS*

| SSH1 | Now I would like to ask you some questions about your brothers and sisters who are born to your biological mother.  Can you please tell me how many sisters were born to your biological mother? This includes sisters who are living with you, sisters who not living with you and those who have died?    INSTRUCTION: assist the respondent with a suggestion to enumerate sisters by listing their first names | 0  NUMBER …………………  DON’T KNOW | SSH6 |
| --- | --- | --- | --- |
| SSH2 | How many of these sisters are no longer with us today (have passed away)? | 0  NUMBER  DK | SSH6 |
| SSH3 | How many of your sisters have passed away since the beginning of 2019? | 0  NUMBER  DK | SSH6 |
| SSH4 | Can you give me the first name for these sisters? | S1 FName:    S2 FName:    S3 FName: |  |
| SSH5 | In which year/month did (NAME) die? | S1 Year:  S1 Month:    S2 Year  S2 Month    S3 Year:  S3 Month: | D LOOP      D LOOP      D LOOP |
| SSH6 | Now I would like to ask you some questions about your brothers who are born to your biological mother.    Can you please tell me how many brothers you have or ever had? Please include brothers who are living with you, sisters who not living with you, and those who have died.    INSTRUCTION: assist the respondent with a suggestion to enumerate brothers by listing their first names | 0  NUMBER  DK |  |
| SSH7 | How many of these brothers are no longer with us today (have passed away)? | 0  NUMBER  DK |  |
| SSH8 | How many of your brothers have passed away since the beginning of 2019? | 0  NUMBER: |  |
| SSH9 | Can you give me the first name for those brothers? | B1 FName:  B2 FName:  B3 FName: |  |
| SSH10 | In which year/month did [NAME] die? | B1 Year:  B1 Month:    B2 Year  B2 Month    B3 Year:  B3 Month: | D LOOP      D LOOP      D LOOP |

  Note: In DRC, we did not collect information on ages at death. In Malawi, we did not collect information disaggregated by sex.

Supplementary Table 3*: Information on siblings collected in each country in the MPS*

|  |  | Burkina Faso | DRC | Malawi |
| --- | --- | --- | --- | --- |
| SSH1 | Total number of siblings | ✓ | ✓ | ✓ |
| SSH1_sex | Total number of siblings disaggregated by sex | ✓ | ✓ |  |
| SSH2 | Total number of deceased siblings | ✓ | ✓ | ✓ |
| SSH2_sex | Total number of deceased siblings disaggregated by sex | ✓ | ✓ |  |
| SSH3 | Total number of siblings who have died since the beginning of 2019 | ✓ | ✓ | ✓ |
| SSH3_sex | Sex of siblings who have died since the beginning of 2019 | ✓ | ✓ | ✓ |
| SSH5  year | Year of death (for deaths in or after 2019) | ✓ | ✓ | ✓ |
| SSH5  month | Month of death (for deaths in or after 2019) | ✓ | ✓ | ✓ |
| SSH5  age at death | Age at death (for deaths in or after 2019) | ✓ |  | ✓ |

Supplementary Table 4*: Information collected in MPS and used for calculating weights in each country*

| Variable | Categories | Burkina Faso | DRC | Malawi |
| --- | --- | --- | --- | --- |
|  |  |  |  |  |
| Education | None  Primary  Secondary and more | ✓ | ✓ | ✓ |
| Age | 15-39 (or 18-39)  40-64 | ✓ | ✓ | ✓ |
| Sex | Male  Female | ✓ | ✓ |  |
| Region | Region of residence | ✓ |  |  |
| Type of place of residence | Urban  Rural | ✓ | ✓ | ✓ |
| Household size | Less than 5 members  5 members and more | ✓ | ✓ | ✓ |
| Electricity | Yes  No | ✓ |  | ✓ |
| Roofing | Improved  Not improved | ✓ |  | ✓ |
| Water | Improved  Not improved | ✓ | ✓ | ✓ |
| Target population | | 2019 census | 2017-2018 MICS | 2015-2016 DHS |

*Note: In the DRC and Malawi MPS, youth aged 15-17 were not eligible to an interview*

Supplementary Table 5*: Composition of the MPS sample in Burkina Faso, by study arm, compared with the population enumerated in the 2019-2020 census*

|  | EHCVM arm (heads of households) | | | 2019-20  census | RDD arm (population aged 15-64 years old) | | | 2019-20 census |
| --- | --- | --- | --- | --- | --- | --- | --- | --- |
| Respondents characteristics | Unweighted | Weighted | |  | Unweighted | Weighted | |  |
|  | % | % | 95% CI | % | % | % | 95% CI | % |
| *Sex* |  | | | |  | | | |
| Men | 76.7 | 84.0 | [82.0-85.7] | 84.0 | 34.3 | 46.2 | [44.7-47.8] | 46.3 |
| Women | 23.3 | 16.0 | [14.3-18.0] | 16.0 | 65.7 | 53.8 | [52.2-55.3] | 53.7 |
| *Age group* |  | | | |  | | | |
| 15-29 | 17.5 | 22.9 | [20.7-25.1] | 22.6 | 41.5 | 43.7 | [42.2-45.3] | 43.7 |
| 30-49 | 52.0 | 49.3 | [46.8-51.9] | 49.1 | 46.8 | 42.2 | [40.7-43.8] | 42.2 |
| 50 &+ | 30.6 | 27.8 | [25.6-30.2] | 28.3 | 11.7 | 14.1 | [13.0-15.2] | 14.1 |
| *Education* |  | | | |  | | | |
| None | 52.0 | 69.5 | [67.1-71.8] | 72.2 | 44.9 | 67.6 | [66.1-69.0] | 69.8 |
| Primary | 20.2 | 11.2 | [9.7-12.9] | 10.0 | 16.5 | 11.5 | [10.5-12.5] | 9.6 |
| Secondary | 21.4 | 13.7 | [12.1-15.6] | 13.5 | 29.5 | 15.7 | [14.6-16.8] | 15.9 |
| Tertiary and higher | 6.4 | 5.6 | [4.5-6.9] | 4.2 | 9.0 | 5.2 | [4.6-5.9] | 3.6 |
| *Marital status* |  | | | |  | | | |
| Married | 83.6 | 83.3 | [81.3-85.1] | 84.2 | 75.4 | 77.5 | [76.2-78.8] | 72.5 |
| Widowed | 5.5 | 5.0 | [4.0-6.2] | 1.1 | 3.3 | 3.6 | [3.1-4.3] | 3.4 |
| Divorced/sep | 0.8 | 0.7 | [0.4-1.3] | 5.6 | 0.8 | 0.7 | [0.5-1.0] | 0.9 |
| Single | 10.1 | 11.0 | [9.5-12.7] | 9.1 | 20.4 | 18.2 | [17.0-19.4] | 23.2 |
| *Type of place of residence* |  | | | |  | | | |
| Ouagadougou | 11.1 | 13.0 | [11.4-14.8] | 14.4 | 15.8 | 11.5 | [10.6-12.4] | 14.5 |
| Bobo-Dioulasso | 6.1 | 3.4 | [2.6-4.4] | 5.0 | 6.7 | 3.8 | [3.3-4.4] | 5.2 |
| Other towns | 40.2 | 13.5 | [12.0-15.3] | 11.9 | 14.3 | 9.3 | [8.5-10.2] | 11.4 |
| Rural areas | 42.6 | 70.1 | [67.7-72.3] | 68.7 | 63.2 | 75.4 | [74.1-76.6] | 69.0 |
| *Region of residence* |  | | | |  | | | |
| Boucle du Mouhoun | 7.1 | 10.5 | [9.0-12.2] | 9.6 | 6.8 | 10.6 | [9.6-11.7] | 9.5 |
| Cascades | 7.4 | 4.3 | [3.4-5.5] | 3.9 | 4.1 | 4.1 | [3.5-4.8] | 4.1 |
| Centre | 15.7 | 16.8 | [14.9-18.8] | 18.4 | 24.3 | 13.4 | [12.5-14.5] | 18.0 |
| Centre-Est | 7.8 | 7.9 | [6.6-9.4] | 7.7 | 6.9 | 8.1 | [7.3-9.1] | 7.4 |
| Centre-Nord | 7.9 | 7.6 | [6.4-9.1] | 7.0 | 9.2 | 8.5 | [7.6-9.4] | 7.3 |
| Centre-Ouest | 6.8 | 7.4 | [6.1-8.9] | 7.8 | 7.6 | 8.3 | [7.5-9.2] | 8.0 |
| Centre-Sud | 5.6 | 4.5 | [3.6-5.7] | 4.0 | 4.3 | 4.6 | [3.9-5.3] | 3.9 |
| Est | 7.1 | 8.5 | [7.2-10.1] | 7.5 | 4.0 | 8.3 | [7.5-9.3] | 7.8 |
| Hauts-Bassins | 12.7 | 12.0 | [10.4-13.7] | 11.8 | 14.2 | 11.0 | [10.1-11.9] | 12.0 |
| Nord | 6.5 | 7.7 | [6.4-9.2] | 7.8 | 7.4 | 8.5 | [7.6-9.4] | 8.1 |
| Plateau-Central | 6.3 | 4.5 | [3.6-5.7] | 4.6 | 6.2 | 4.8 | [4.2-5.6] | 4.7 |
| Sahel | 2.9 | 3.8 | [2.9-4.9] | 5.3 | 1.7 | 5.4 | [4.7-6.2] | 4.6 |
| Sud-Ouest | 6.2 | 4.4 | [3.5-5.6] | 4.8 | 3.4 | 4.3 | [3.7-5.0] | 4.6 |
| *Household size* |  | | | |  | | | |
| 1-4 members | 21.5 | 32.9 | [30.5-35.3] | 49.4 | 28.5 | 39.2 | [37.6-40.7] | 49.4 |
| 5-8 members | 43.0 | 37.4 | [34.9-39.9] | 36.5 | 41.9 | 33.7 | [32.3-35.3] | 36.5 |
| 9+ | 35.4 | 29.7 | [27.4-32.1] | 14.1 | 29.6 | 27.1 | [25.7-28.6] | 14.1 |

Note: The percentages in the columns referring to the census are specific to each arm because the EHCVM study arm is compared to the population of heads of households in the census, while the RDD sample is compared to the population aged 15-64.

Supplementary Table 6**:** *Composition of the MPS sample in DRC, by province, compared with the population enumerated in the MICS 2017-2018 survey*

| Respondents characteristics | Kinshasa | | | | North Kivu | | | |
| --- | --- | --- | --- | --- | --- | --- | --- | --- |
|  | Unweighted | Weighted | | MICS-2017/18 | Unweighted | Weighted | | MICS-2017/18 |
|  | % | % | 95% CI | % | % | Percentage | 95% CI | % |
| *Sex* |  | | | | | | | |
| Female | 41.9 | 46.3 | [44.8-47.9] | 54.3 | 37.7 | 35.6 | [33.8-37.5] | 56.1 |
| Male | 58.1 | 53.7 | [52.1-55.2] | 45.7 | 62.3 | 64.4 | [62.5-66.2] | 43.9 |
| *Age group* |  | | | | | | | |
| 18-39 | 69.3 | 63.4 | [61.9-64.9] | 79.6 | 79.6 | 66.3 | [64.5-68.1] | 79.7 |
| 40-64 | 30.7 | 36.6 | [35.1-38.1] | 22.2 | 20.4 | 33.7 | [31.9-35.5] | 20.3 |
| *Education* |  | | | | | | | |
| None/primary | 2.6 | 5.3 | [4.5-6.1] | 11.8 | 19.1 | 37.6 | [35.8-39.5] | 41.7 |
| Secondary | 40.3 | 71.4 | [70.2-72.5] | 63.5 | 52.0 | 45.1 | [43.2-47.0] | 43.7 |
| Superior | 57.1 | 23.4 | [22.5-24.3] | 24.6 | 28.9 | 17.3 | [16.0-18.7] | 14.6 |
| *Marital status* |  | | | | | | | |
| Married/Cohabiting | 53.3 | 54.3 | [52.8-55.8] |  | 56.0 | 65.0 | [63.2-66.8] |  |
| Widowed | 1.9 | 3.1 | [2.6-3.8] |  | 0.9 | 1.4 | [1.0-1.9] |  |
| Divorced/Separated | 3.9 | 5.2 | [4.5-6.0] |  | 2.0 | 2.7 | [2.1-3.5] |  |
| Single | 40.8 | 37.3 | [35.9-38.8] |  | 41.1 | 30.9 | [29.1-32.7] |  |
| *Household assets* |  | | | | | | | |
| No | 0.7 | 1.0 | [0.7-1.3] | 0.0 | 18.6 | 38.5 | [36.6-40.4] | 4.1 |
| Yes | 99.3 | 99.0 | [98.7-99.3] | 100.0 | 81.4 | 61.5 | [59.6-63.4] | 95.9 |
| *Type of place of residence* |  | | | | | | | |
| Urban | 95.2 | 93.5 | [92.6-94.2] | 100.0 | 91.1 | 65.2 | [63.1-67.3] | 41.0 |
| Rural | 4.8 | 6.5 | [5.8-7.4] | 0.0 | 8.9 | 34.8 | [32.7-36.9] | 59.0 |
| *Household size* |  | | | | | | | |
| 1-4 members | 32.8 | 29.3 | [28.0-30.7] | 42.3 | 20.3 | 18.1 | [16.7-19.7] | 32.0 |
| 5-8 members | 49.2 | 50.7 | [49.2-52.3] | 45.0 | 47.1 | 45.8 | [43.9-47.8] | 50.5 |
| 9+ members | 18.1 | 19.9 | [18.7-21.2] | 12.7 | 32.5 | 36.0 | [34.2-37.9] | 17.5 |

Note: The sample in each region for RaMMPS survey is compared to the population aged 18-64 years old of these same regions in the MICS 2017-2018 survey

Supplementary Table 7**:** *Composition of the MPS sample in Malawi, compared with the population enumerated in the 2018 census*

| Respondents characteristics | Unweighted | Weighted | | 2018  census |
| --- | --- | --- | --- | --- |
|  | % | % | 95% CI | % |
| *Sex* |  | | | |
| Female | 47.5 | 46.9 | [45.6-48.2] | 46.9 |
| Male | 52.5 | 53.1 | [51.8-54.4] | 53.2 |
| *Age group* |  | | | |
| 18-49 | 92.3 | 85.1 | [84.1-86.1] | 77.3 |
| 50+ | 7.7 | 14.9 | [13.9-15.9] | 22.7 |
| *Education* |  | | | |
| None/primary | 22.5 | 44.3 | [43.0-45.6] | 72.7 |
| Secondary | 50.9 | 49.1 | [47.8-50.4] | 23.4 |
| Superior | 26.6 | 6.6 | [5.9-7.3] | 3.9 |
| *Marital status* |  | | | |
| Married/Cohabiting | 58.0 | 64.8 | [63.6-66.0] | 66.1 |
| Widowed | 2.4 | 3.5 | [3.0-4.0] | 7.2 |
| Divorced/Separated | 7.2 | 8.8 | [8.1-9.6] | 7.9 |
| Single | 32.4 | 22.9 | [21.8-23.9] | 18.8 |
| *Type of place of residence* |  | | | |
| Urban | 26.0 | 18.0 | [17.1-19.0] | 16.9 |
| Rural | 74.0 | 82.0 | [81.0-82.9] | 83.1 |
| *Region* |  | | | |
| Central | 39.9 | 40.7 | [39.4-42.0] | 43.1 |
| Northern | 15.5 | 12.9 | [12.1-13.9] | 12.2 |
| Southern | 44.6 | 46.4 | [45.1-47.7] | 44.7 |
| *Household size* |  | | | |
| 1-4 members | 39.3 | 34.4 | [33.2-35.7] | 92.0 |
| 5-8 members | 51.8 | 56.4 | [55.1-57.7] | 7.8 |
| 9+ members | 8.9 | 9.2 | [8.5-9.9] | 0.2 |

Note: The sample is compared to the population aged 18-64 years old in Malawi

Supplementary Table 8*: Estimates of the probability _35_q_15_ over the period 0-3 years before data collection, by source and sex of respondent and 95% confidence intervals per country (‱), based on partial imputation*

| Country |  |  | Male mortality | Female mortality | Both sexes combined | Relative difference with DHS (%) | | | Relative difference with  WPP (%) | | |
| --- | --- | --- | --- | --- | --- | --- | --- | --- | --- | --- | --- |
|  | source | Sex of resp | _35_q_15_  [IC] | _35_q_15_  [IC] | _35_q_15_  [IC] | Male mortality | Female mortality | Both sexes | Male mortality | Female mortality | Both sexes |
| Burkina Faso | Both arms combined | Male | 85  [66 – 103] | 58  [42 – 74] | 71  [62.1 – 79.1] | -12 | -13 | -14 | -52 | -58 | -55 |
|  |  | Female | 78  [59 – 93] | 65  [49 – 80] |  | -20 | -3 |  | -56 | -53 |  |
|  |  | Both | 81  [68 – 98] | 60  [46 – 67] |  | -16 | -10 |  | -54 | -57 |  |
|  | EHCVM | Male | 101  [70 – 132] | 57  [32 – 82] | 69  [55.6 – 81.9] | 4 | -15 | -17 | -43 | -59 | -56 |
|  |  | Female | 59  [34 – 83] | 58  [31 – 85] |  | -39 | -13 |  | -67 | -58 |  |
|  |  | Both | 82  [67 – 107] | 55  [38 – 67] |  | -15 | -18 |  | -54 | -60 |  |
|  | RDD | Male | 72  [53 – 90] | 58  [40 – 77] | 71  [60.6 – 81.9] | -26 | -13 | -14 | -60 | -58 | -55 |
|  |  | Female | 88  [63 – 112] | 70  [49 – 90] |  | -9 | 4 |  | -51 | -49 |  |
|  |  | Both | 79  [66 – 99] | 64  [45 – 75] |  | -19 | -4 |  | -56 | -54 |  |
|  | DHS-2021 | | 97  [83 – 110] | 67  [55 – 80] | 83  [73.4 – 92.1] |  |  |  |  |  |  |
|  | WPP | | 178 | 138 | 158 |  |  |  |  |  |  |
|  | | | | | | | | | | | |
| Kinshasa | Male | | 46  [27 – 64] | 67  [40 – 93] | 57  [45.3 – 68.0] | -70 | -25 | -53 | -63 | -43 | -53 |
|  | Female | | 72  [43 – 99] | 35  [12 – 57] |  | -53 | -61 |  | -42 | -70 |  |
|  | Both | | 57  [40 – 73] | 52  [35 – 70] |  | -62 | -42 |  | -54 | -56 |  |
| North-Kivu | Male | | 71  [41 – 99] | 40  [21 – 59] | 47  [36.2 – 58.0] | -51 | -74 | -68 | -25 | -53 | -48 |
|  | Female | | 57  [28 – 86] | 25  [6 – 43] |  | -61 | -72 |  | -40 | -71 |  |
|  | Both | | 66  [44 – 86] | 35  [21 – 49] |  | -55 | -61 |  | -31 | -59 |  |
| Kinshasa | DHS-2013 | | 151  [103 – 197] | 90  [55 – 124] | 122  [90.6 – 153.0] |  |  |  |  |  |  |
|  | WPP | | 123 | 118 | 121 |  |  |  |  |  |  |
| North-Kivu | DHS-2013 | | 145  [85 – 202] | 154  [83 – 220] | 149  [106.7 – 90.3] |  |  |  |  |  |  |
|  | WPP | | 95 | 85 | 90 |  |  |  |  |  |  |
|  | | | | | | | | | | | |
| Malawi | Male | |  | | 147  [120.5 – 72.9] |  | | -16 |  | | -16 |
|  | Female | |  | | 107  [78.7 – 136.1] |  | | -38 |  | | -38 |
|  | Both | |  | | 131  [111.4 – 50.0] |  | | -25 |  | | -25 |
|  | DHS-2015 | | 204  [183.3 – 24.3] | 144  [126.4 – 161.7] | 175  [161.1 – 87.8] |  |  |  |  |  |  |
|  | WPP | | 222 | 130 | 176 |  |  |  |  |  |  |

*Note: EHCVM : Enquête Harmonisée sur les Conditions de Vie des Ménages ; RDD : Random Digit Dialing ; WPP : World Population Prospects ; DHS : Demographic and Health Survey*

Supplementary Table 9*: Estimates of the probability _35_q_15_ over the period 0-3 years before data collection, by source and sex of respondent and 95% confidence intervals per country (deaths before age 50 for 1000 adolescents aged 15), based on complete imputation*

| Country |  |  | Men | Women | Both | Relative difference with DHS (%) | | | Relative difference with  WPP (%) | | |
| --- | --- | --- | --- | --- | --- | --- | --- | --- | --- | --- | --- |
|  | ***source*** | ***Sex of resp*** | ***35q15***  ***[IC]*** | ***35q15***  ***[IC]*** | ***35q15***  ***[IC]*** | ***Men*** | ***Women*** | ***Both*** | ***Men*** | ***Women*** | ***Both*** |
| Burkina Faso | Both arms combined | Male | 161  [138 - 183] | 102  [82 - 121] | 154  [143 – 166] | 66 | 52 | 85 | -10 | -26 | -3 |
|  |  | Female | 223  [194 - 252] | 130  [108 - 152] |  | 130 | 94 |  | 25 | -6 |  |
|  |  | Both | 190  [174 - 207] | 115  [102 - 129] |  | 96 | 72 |  | 7 | -17 |  |
|  | EHCVM | Male | 168  [128 - 206] | 111  [81 - 139] | 165  [148 – 183] | 73 | 66 | 99 | -6 | -20 | 4 |
|  |  | Female | 246  [198 - 288] | 141  [102 - 177] |  | 154 | 110 |  | 38 | 2 |  |
|  |  | Both | 205  [176 - 233] | 124  [102 - 144] |  | 111 | 85 |  | 15 | -10 |  |
|  | RDD | Male | 155  [125 - 185] | 99  [69 - 128] | 148  [132 – 163] | 60 | 48 | 78 | -13 | -28 | -6 |
|  |  | Female | 210  [171 - 246] | 124  [97 - 151] |  | 116 | 85 |  | 18 | -10 |  |
|  |  | Both | 180  [157 - 203] | 111  [92 - 130] |  | 86 | 66 |  | 1 | -20 |  |
|  | DHS-2021 | | 97  [83 - 110] | 67  [55 - 80] | 83  [73.4 – 92.1] |  |  |  |  |  |  |
|  | WPP | | 178 | 138 | 158 |  |  |  |  |  |  |
|  | | | | | | | | | | | |
| Kinshasa | Male | | 65  [42 - 88] | 47  [26 - 68] | 57  [45 – 68] | -57 | -47 | -53 | -47 | -60 | -53 |
|  | Female | | 61  [35 - 86] | 53  [27 - 78] |  | -60 | -41 |  | -50 | -55 |  |
|  | Both | | 63  [46 - 80] | 50  [33 - 66] |  | -58 | -44 |  | -49 | -58 |  |
| North-Kivu | Male | | 71  [42 - 99] | 66  [38 - 93] | 72  [57 – 87] | -51 | -57 | -52 | -25 | -22 | -20 |
|  | Female | | 89  [46 - 131] | 73  [36 - 108] |  | -38 | -53 |  | -6 | -14 |  |
|  | Both | | 76  [53 - 98] | 68  [45 - 91] |  | -48 | -56 |  | -20 | -20 |  |
| Kinshasa | DHS-2013 | | 151  [103 - 197] | 90  [55 - 124] | 122  [90.6 – 153.0] |  |  |  |  |  |  |
|  | WPP | | 123 | 118 | 121 |  |  |  |  |  |  |
| North-Kivu | DHS-2013 | | 145  [85 - 202] | 154  [83 - 220] | 149  [106.7 – 90.3] |  |  |  |  |  |  |
|  | WPP | | 95 | 85 | 90 |  |  |  |  |  |  |
|  | | | | | | | | | | | |
| Malawi | Male | |  | | 165  [135 – 194] |  | | -6 |  | | -6 |
|  | Female | |  | | 193  [148 – 237] |  | | 10 |  | | 10 |
|  | Both | |  | | 177  [152 – 200] |  | | 1 |  | | 1 |
|  | DHS-2015 | | 204  [183 – 224] | 144  [126 – 162] | 175  [161 – 187] |  |  |  |  |  |  |
|  | WPP | | 222 | 130 | 176 |  |  |  |  |  |  |

*Note: EHCVM : Enquête Harmonisée sur les Conditions de Vie des Ménages ; RDD : Random Digit Dialing ; WPP : World Population Prospects ; DHS : Demographic and Health Survey*

### A.4.2 Additional figures

Supplementary Figure 4: *Composition of the MPS samples and comparison with nation-wide censuses or surveys in Burkina Faso, Malawi and DRC*


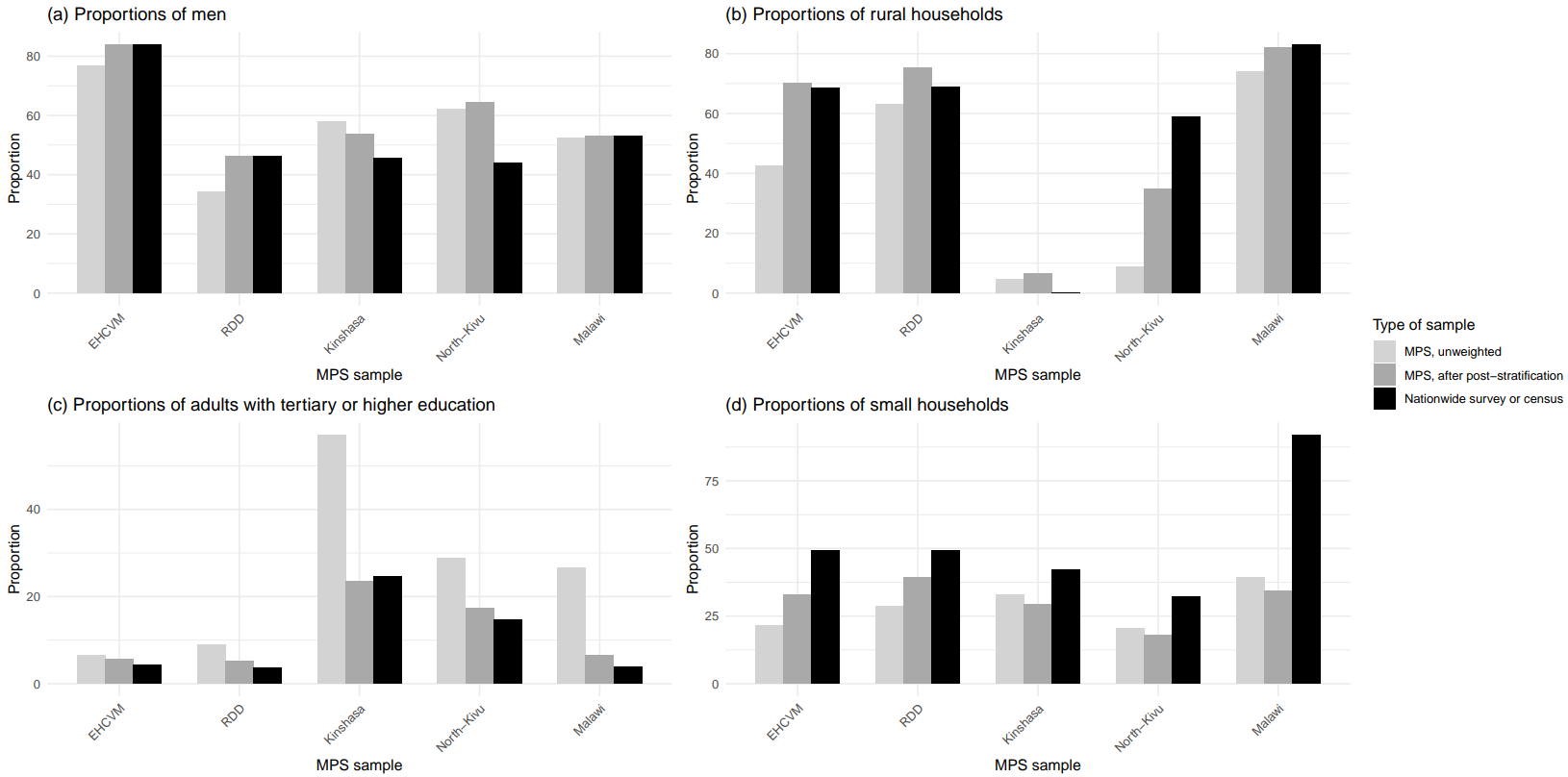


Supplementary Figure 5*. Proportions of surviving siblings at the time of the survey, by age group of respondent and sex of siblings in MPS and DHS, in Burkina Faso and DRC*


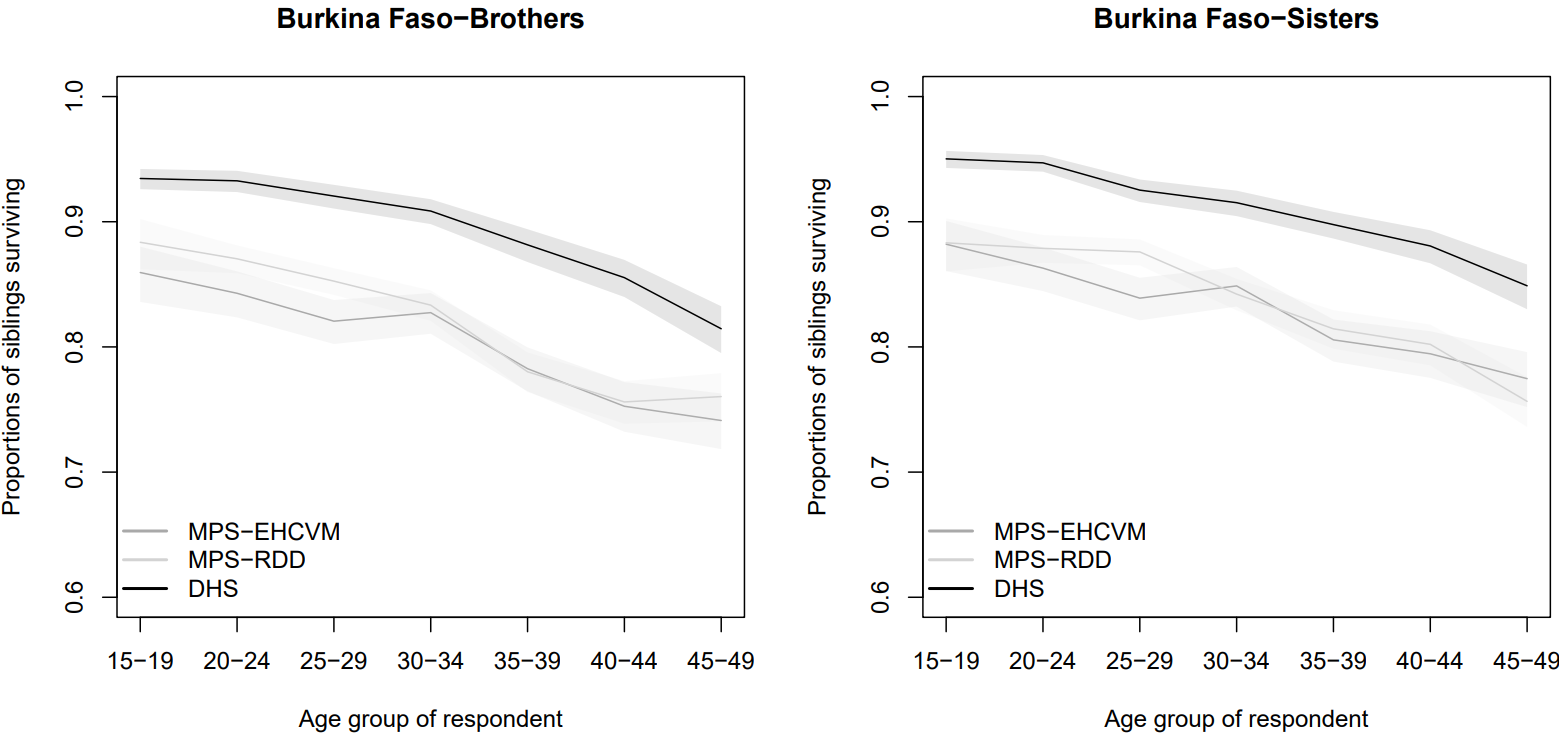

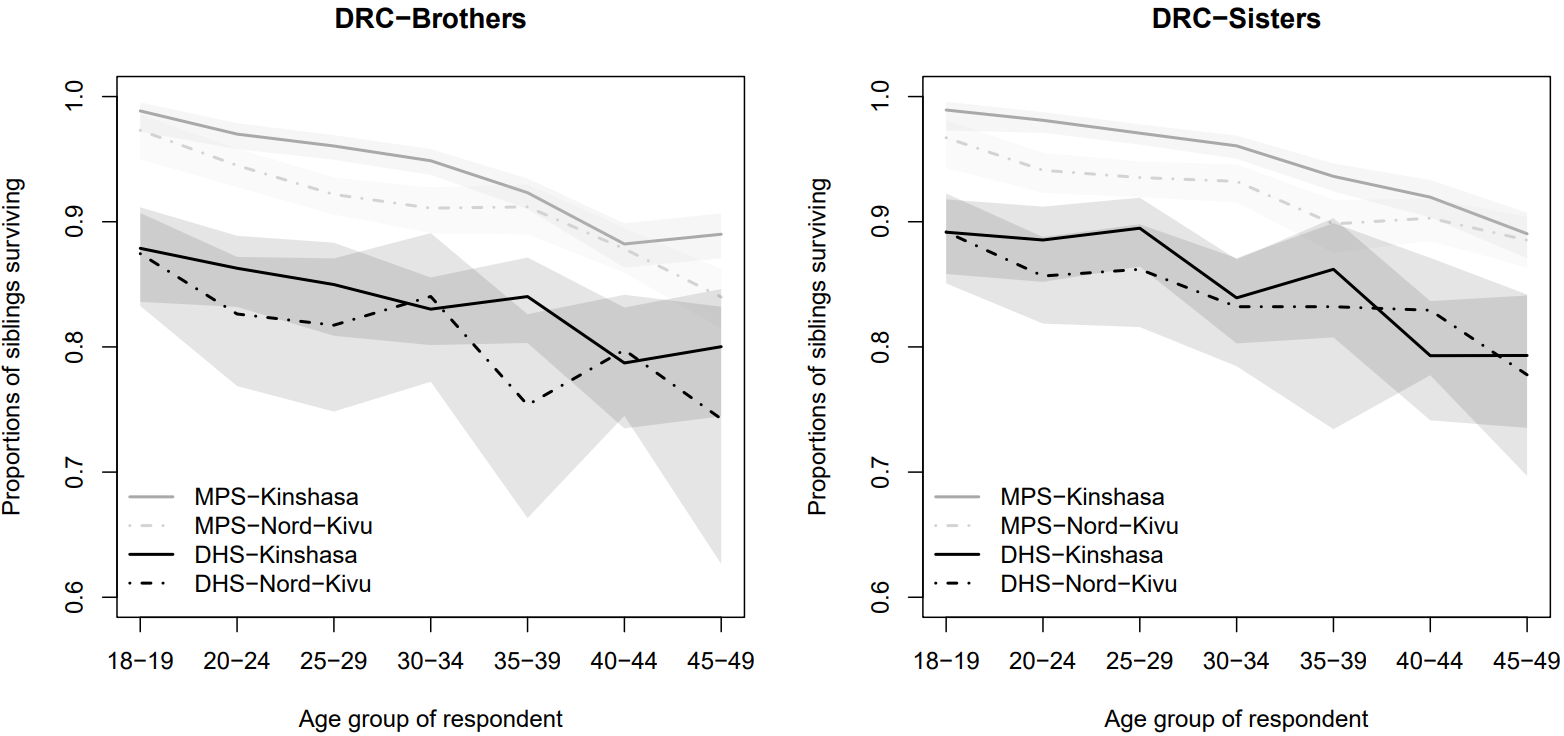


Supplementary Figure 6*. Proportions of recent deaths among all deceased siblings by age group of respondent and sex of sibling (last 3 years in DHS and since January 2019 in MPS) in Burkina Faso and DRC*


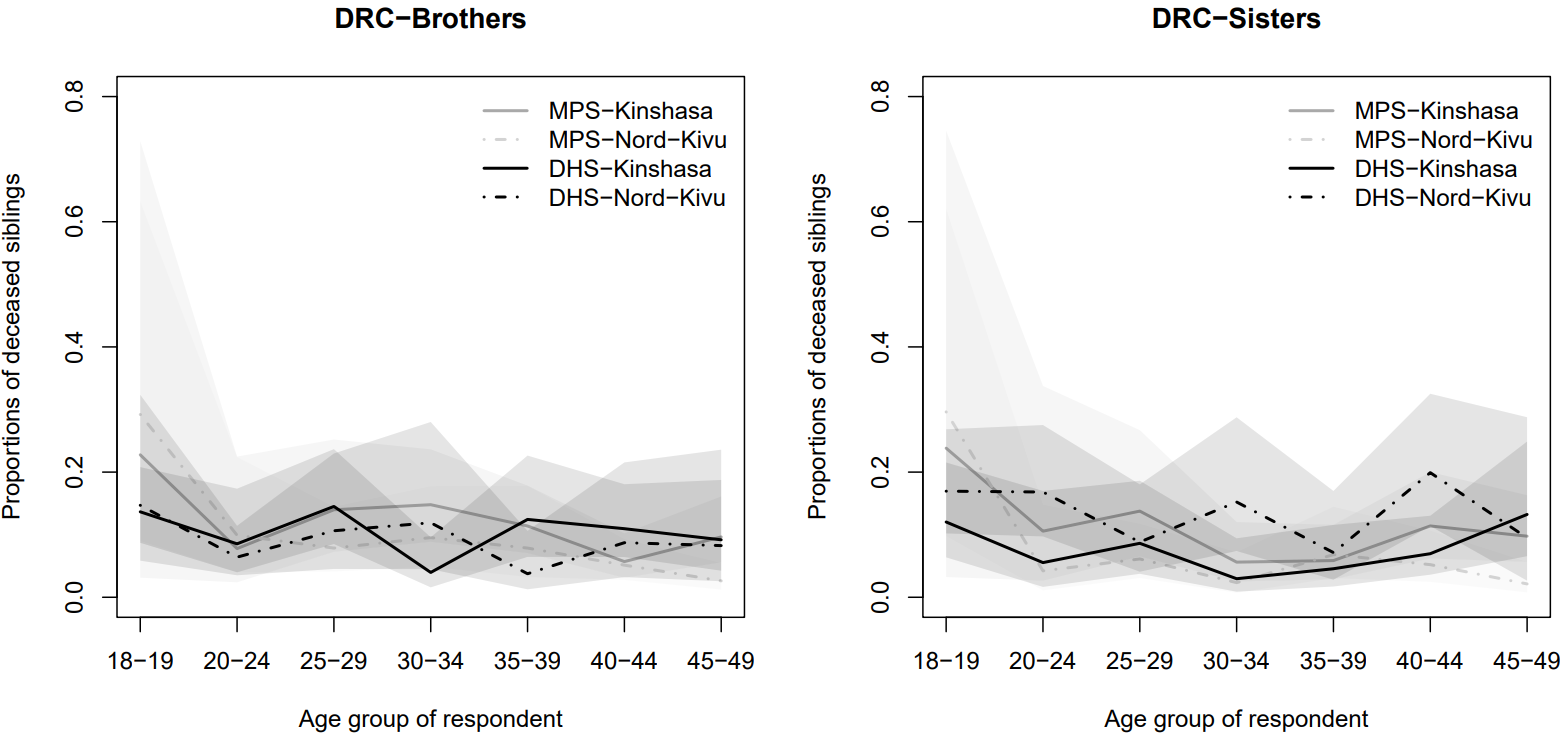

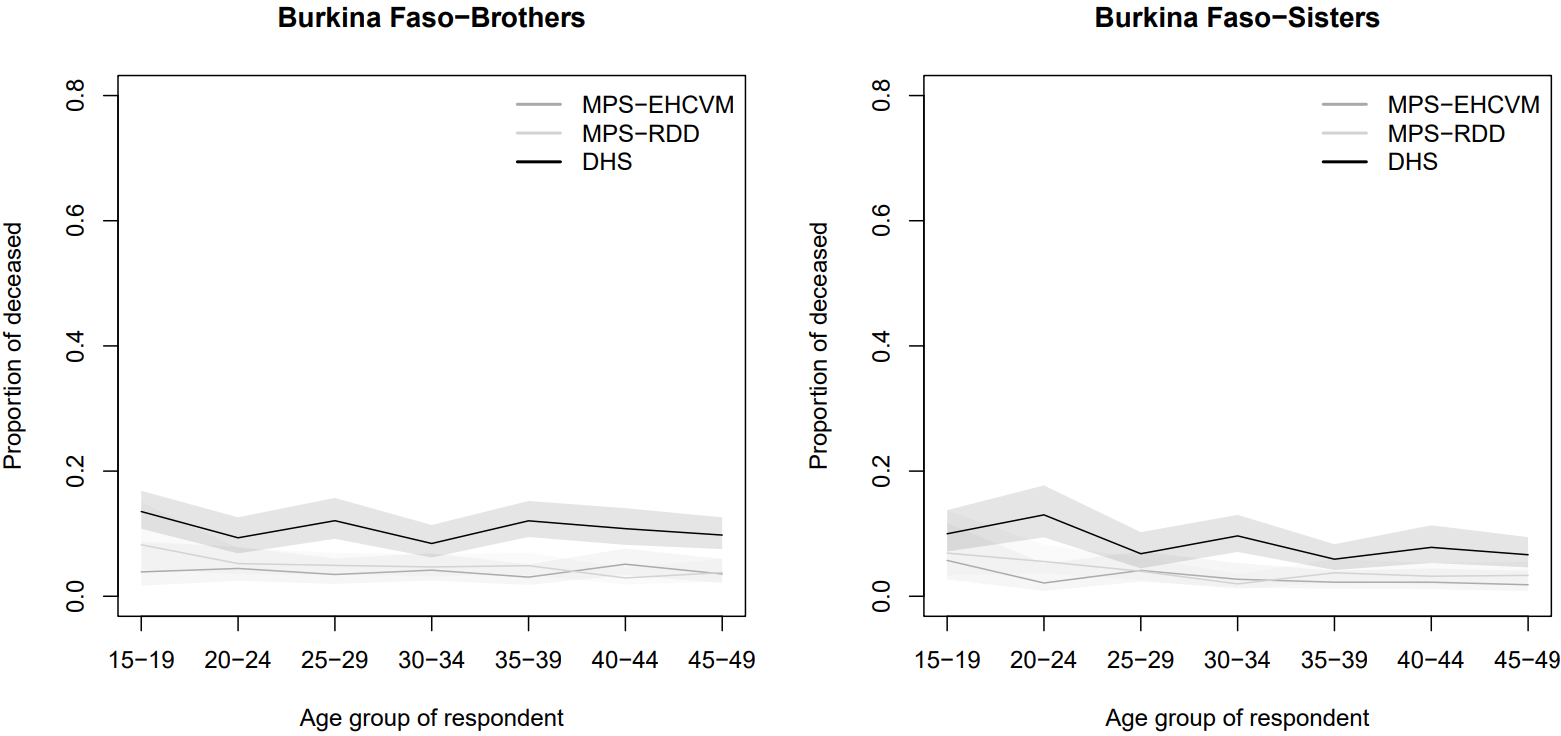


Supplementary Figure 7*: Trends in adult mortality (_35_q_15_) according to SSH in the MPS survey (using partial imputation) or DHS surveys and in the World Population Prospects*


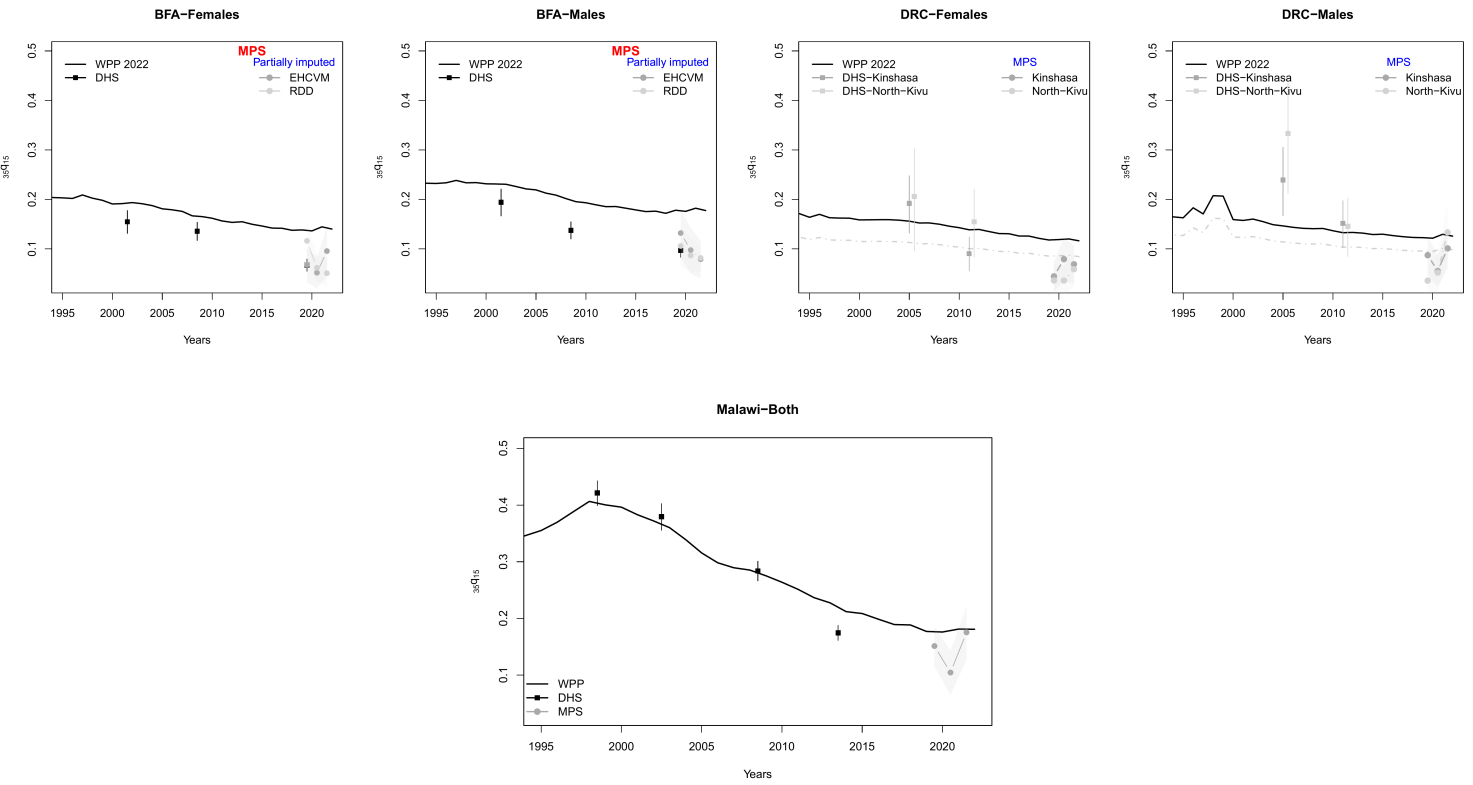


Supplementary Figure 8*:* Age-at-death distribution among adult siblings who died in the last 3 years


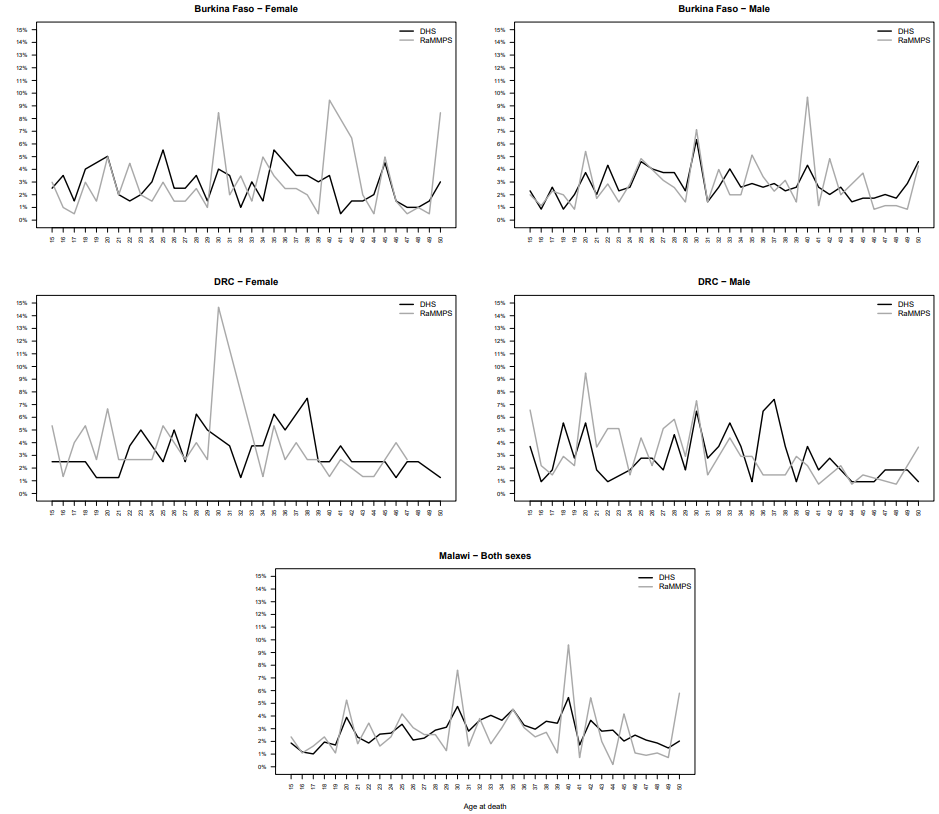

Supplement: online supplemental file 1 [file bmjgh-10-11-s001.docx]
